# The modular network structure of the mutational landscape of Acute Myeloid Leukemia

**DOI:** 10.1371/journal.pone.0202926

**Published:** 2018-10-10

**Authors:** Mariam Ibáñez, José Carbonell-Caballero, Esperanza Such, Luz García-Alonso, Alessandro Liquori, María López-Pavía, Marta Llop, Carmen Alonso, Eva Barragán, Inés Gómez-Seguí, Alexander Neef, David Hervás, Pau Montesinos, Guillermo Sanz, Miguel Angel Sanz, Joaquín Dopazo, José Cervera

**Affiliations:** 1 Hematology Service, Hospital Universitario y Politécnico La Fe, Valencia, Spain; 2 Centro de Investigacion Biomédica en Red de Cáncer (CIBERONC), Instituto Carlos III, Madrid, Spain; 3 Departamento de Ciencias Biomédicas, Facultad de Ciencias de la Salud, Universidad CEU Cardenal Herrera, Valencia, Spain; 4 ProCURE, Catalan Institute of Oncology, Bellvitge Institute for Biomedical Research (IDIBELL), L’Hospitalet del Llobregat, Barcelona, Spain; 5 European Molecular Biology Laboratory—European Bioinformatics Institute, Wellcome Genome Campus, Cambridge, United Kingdom; 6 Department of Medical Pathology, Hospital Universitario La Fe, Valencia, Spain; 7 Hematology Service, Hospital Arnau de Villanoba, Valencia, Spain; 8 Biostatistics Unit, IIS La Fe Valencia, Spain; 9 Functional Genomics Node, Spanish National Institute of Bioinformatics at CIPF, Valencia, Spain; 10 Bioinformatics of Rare Diseases (BIER), CIBER de Enfermedades Raras (CIBERER), Valencia, Spain; 11 Clinical Bioinformatics Area, Fundación Progreso y Salud (FPS), CDCA, Hospital Virgen del Rocio, Sevilla, Spain; 12 Genetics Unit, Hospital Universitario y Politécnico La Fe, Valencia, Spain; Emory University, UNITED STATES

## Abstract

Acute myeloid leukemia (AML) is associated with the sequential accumulation of acquired genetic alterations. Although at diagnosis cytogenetic alterations are frequent in AML, roughly 50% of patients present an apparently normal karyotype (NK), leading to a highly heterogeneous prognosis. Due to this significant heterogeneity, it has been suggested that different molecular mechanisms may trigger the disease with diverse prognostic implications. We performed whole-exome sequencing (WES) of tumor-normal matched samples of *de novo* AML-NK patients lacking mutations in *NPM1*, *CEBPA* or *FLT3*-ITD to identify new gene mutations with potential prognostic and therapeutic relevance to patients with AML. Novel candidate-genes, together with others previously described, were targeted resequenced in an independent cohort of 100 *de novo* AML patients classified in the cytogenetic intermediate-risk (IR) category. A mean of 4.89 mutations per sample were detected in 73 genes, 35 of which were mutated in more than one patient. After a network enrichment analysis, we defined a single *in silico* model and established a set of seed-genes that may trigger leukemogenesis in patients with normal karyotype. The high heterogeneity of gene mutations observed in AML patients suggested that a specific alteration could not be as essential as the interaction of deregulated pathways.

## Introduction

Acute myeloid leukemia (AML) is associated with the sequential accumulation of acquired genetic alterations and epigenetic changes in hematopoietic stem cells that alter processes involved in proliferation, differentiation and self-renewal. At diagnosis, cytogenetic alterations are frequent in AML patients [1,2]. Nevertheless, at least 50% of patients present an apparently normal karyotype (NK) being its prognosis highly heterogeneous. These patients are consequently classified in the cytogenetic intermediate-risk (IR) category. However, their significant heterogeneity suggests that there are different molecular mechanisms involved in the development of the disease as well as related to different prognostic implications [3–5]. The recent progress of next-generation sequencing (NGS) technologies has allowed for the identification of a growing number of novel mutations in leukaemias [6–9]. Their application has led to broaden the list of genes that seems to be involved in the pathogenesis of such disorders. Consequently, it has reflected their complexity and described patterns of cooperation and exclusion between different genes and cellular pathways involved in the mechanisms of leucemogenesis. In recent years, The Cancer Genome Atlas (TCGA) has collected data from exomic or genomic sequencing, expression, methylation, and genotyping of 200 adult patients with AML, identifying 23 different frequently mutated genes [10]. These genes were grouped into 8 different categories by their cellular function, detecting at least one mutation per group in 99% of the studied AML patients. Recently, Papaemmanuil et al. defined 3 additional molecular subgroups in AML patients with prognostic implications [11]. However, clinicians only rely on the presence of alterations of 3 well established molecular markers, *NPM1*, *CEBPA* or *FLT3*-ITD, for the diagnosis classification of patients with AML-IR [12].

To extend our knowledge on subtle genetic alterations involved in AML-NK, in this study, we have performed whole-exome sequencing (WES) of tumor-normal matched samples on a selected discovery cohort of *de novo* AML patients. To this aim, we studied samples of leukemia cells from adults under the age of 60 years who lacked cytogenetic abnormalities and well known molecular features that is, wild-type *NPM1*, *CEBPA*, and *FLT3*–ITD. Genes identified from this analysis were validated on an independent cohort by targeted resequencing. Finally, network analysis [13] was performed *in silico* to assess their putative role as driver genes.

## Material and methods summary

We performed whole-exome sequencing (WES) of tumor-normal matched samples from 7 *de novo* AML-NK patients without mutations in *NPM1*, *CEBPA* and *FLT3*–ITD (“discovery cohort”). WES data were analyzed using an in-house bioinformatics pipeline [14] to compare the coding sequence of matched samples, filter out germline variants and identify somatically acquired deleterious changes. The variants detected were confirmed in both samples of each patient by targeted sequencing using an Ion AmpliSeq™ analysed in an Ion Proton™ System. By using the SureDesign Tool (Agilent) for NGS we developed a custom design targeting hotspot regions of 55 genes found mutated in the discovery cohort. Furthermore, complete coding sequence of extra 32 genes (reported to be mutated in at least 2% of patients from previous AML studies [10]) were also included. This design was tested in 100 additional *de novo* AML-IR patients (“validation cohort”). Variants were selected according to VAF≥1%, its absence in the healthy population (UCSC Common SNPs; MAF < 0.01) and its putative effect on the protein (excluding synonymous mutations). Finally, network enrichment analysis [13–16] was used to assess the candidate genes on the basis of their connectivity, mutational recurrence and co-occurrence and cancer related functionalities.

This study was approved by the Research Ethics Board of IISLAFE (No.2012/0175) and informed consent in accordance with the Declaration of Helsinki was obtained before taking sample for genetic and genomic research.

Further Material and Methods details are provided in the Extended Experimental Procedures at S1 File.

## Results

### Whole exome sequencing

We characterized a discovery cohort of 7 AML-NK *de novo* patients from whom paired diagnosis and complete remission DNA samples were available. Clinical characteristics of the patients at diagnosis are summarized in S1 Table. The target coverage average in both diagnostic and remission samples and the mean of reads by genomes was 228.97(±125.78)x and 32944(±4192), respectively (S2 and S3 Tables). A total of 402,412 variant positions were detected in all sequenced exons from all the samples. After an accurate filtering of the sequencing data [14], a total of 102 candidate somatic variants (94 missense SNVs and 8 small indels) were found (S3 Table). The distribution of the number of variants per exome showed an average of 30 mutations per sample (range 22–37). Recurrent mutations or common mutated genes were not observed within the seven cases. Targeted resequencing of both samples of the discovery cohort confirmed 64/102 deleterious somatic variants in 55 genes. Among them, 57 were missense mutations and 8 indels, which stood for an average of 9.2 mutations by sample (range 3–22). Of these 55 candidate genes, 26 were previously reported as mutated in AML patients in previous studies [10, 11]. Detailed information is provided at S3 Table.

### Frequency and distribution of 87 genes in a validation cohort of AML patients

**Fig 1:**
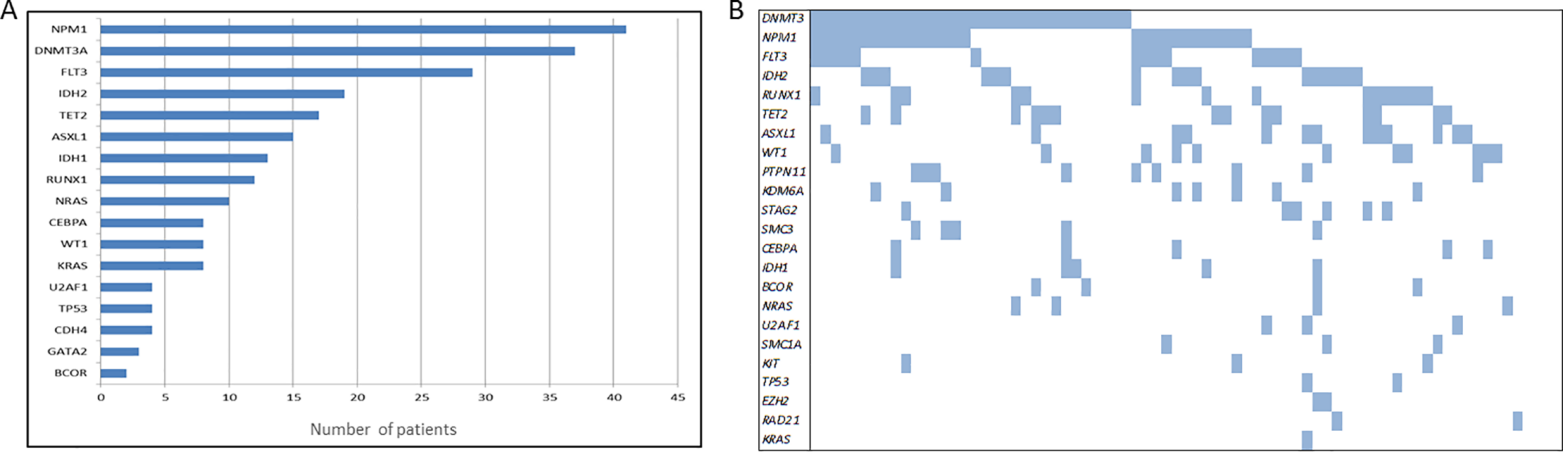
Distribution of selected mutations along the different affected genes of the validation cohort. A) Number of mutated samples by gene according to the described mutation filtering protocol. Only recurrent genes were included. B) Co-occurrence of all somatic mutations.

We further examined the mutation frequency of our candidate genes in an independent validation cohort of 100 *de novo* AML patients with intermediate cytogenetic risk. Details of the validation cohort are provided in S4 Table. Overall, mean target coverage was 260±106x, with an absolute median coverage of 220x at positions where mutations were identified. After processing the raw sequencing data with the procedures described in [14] we identified a total of 158 high-confidence different variants. Mutations were predominantly missense substitutions (53%), frameshift indels (34%), and nonframeshift indels (6%), in splice site regions (4%), nonsenses (2%), or ncRNA (1%) (S5 Table). A mean of 4.89 mutations per sample (range 0–10) were detected. Variants were detected affecting 73 different genes, being 28 of these variants concurrently harboured in more than one patient. Among the mutated genes, 35 showed mutations in at least 7% of the patients (range 7–34%), being the most frequent mutated genes *NPM1*, *DNMT3A*, *FLT3*, *TET2* and *ASXL1* (S5 Table). *In silico* analysis showed that most of the observed mutations were reported in the COSMIC database as mutations implicated in cancer. In addition, as we expected, recurrent hotspots previously reported were detected in genes such as *DNMT3A*, *NPM1*, *NRAS*, *KRAS*, *FLT3*, *IDH2* and *IDH1* (S5 Table). Finally, we analysed the co-occurrence of the identified mutations in our validation cohort, and identified that patients with *DNMT3A*, *NPM1* and *FLT3* did not usually harboured other mutations. In contrast, patients with *DNMT3A* and *NPM1* appeared simultaneously with genes such as *IDH2*, *PTPN11* or *RUNX1*. Finally, patients lacking mutations in *DNMT3A* showed a pattern that included a miscellany of altered genes (Fig 1).

**Fig 2:**
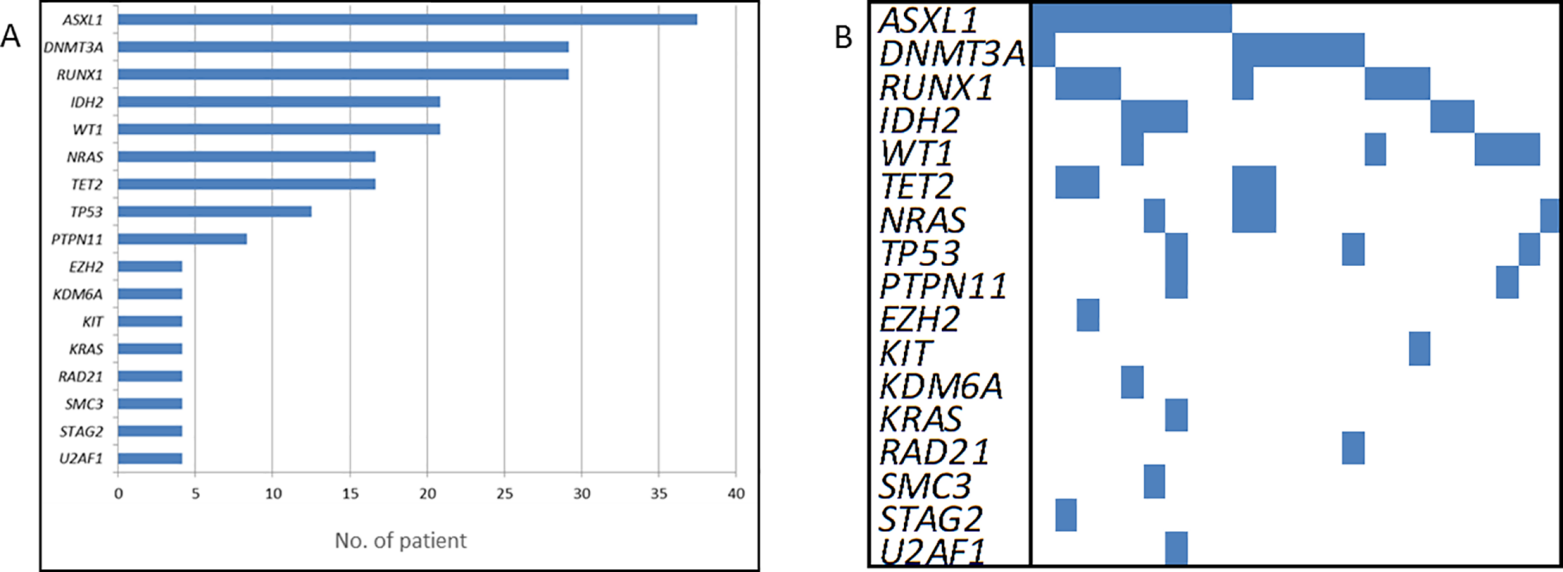
Distribution of selected mutations along the different affected genes among validation cohort with same molecular features than the discovery cohort. A) Number of mutated samples by gene according to the described mutation filtering protocol. Only recurrent genes were included. B) Co-occurrence of all somatic mutations.

We found somatic mutations with deleterious effect in 100% of patients of the “validation cohort” (n = 24) carrying the same molecular features (*i*.*e*. without mutations in *NPM1*, *CEBPA* and *FLT3*–ITD) than the “discovery cohort”. Among them, 75% carried mutations in *ASXL1*, *DNMT3A* and/or *RUNX1*. Other genes such as *DNAH9*, *IDH2*, *WT1*, *DNAH8*, *NRAS* or *TET2* were also recurrently mutated within this set of patients (Fig 2A). In addition, although *DNMT3A* mutations tend to appear as isolated alterations, *ASXL1* and *RUNX1* mutations mainly co-occur among them and with mutations in other genes (Fig 2B). In both cases, given the nature of these genes, the epigenetic regulation might be compromised. Moreover, we extended our analysis to the mutations described in AML patients from the TCGA cohort with the same molecular features (n = 20). Within TCGA patients, the most frequently mutated genes were *DNMT3A*, *RUNX1*, *IDH2*, *TET2*, and *NRAS*. Taking into account both sets of patients, we obtained an input of 44 samples with normal karyotype lacking mutations in *NPM1*, *CEBPA*, and *FLT3-*ITD, who harbor mutations in: *DNMT3A* (32%), *RUNX1* (28%), *ASXL1* (23%), *IDH2* (21%), *NRAS* (17%), and *TET2* (17%) genes.

### Candidate gene prioritization

**Fig 3:**
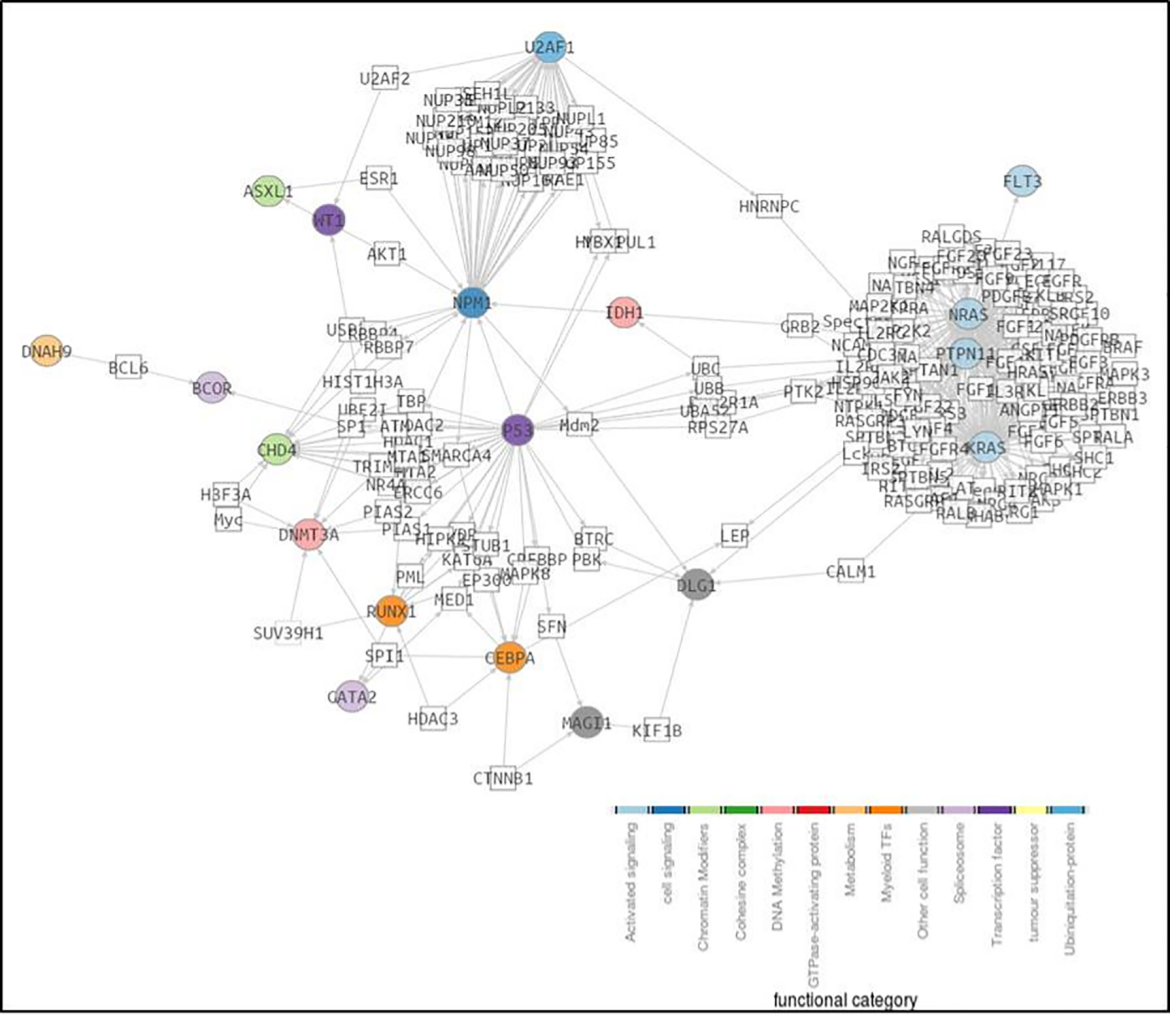
Network-based analysis (SNOW; Babelomics) applied to 28 selected genes. The network was complemented with the co-occurrence relationships, in order to summarize the two kind of significant results. Significant network-based analysis genes are coloured depending on their biological role and circle shaped. Intermediate genes were painted in white and square shaped. While grey edges represent protein-protein interaction, relationships, broad orange dashed lines describe significant co-occurrences.

**Fig 4:**
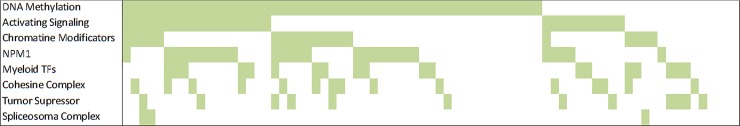
Distribution of mutations according to their functional category among the validation cohort.

Focusing on the deleterious variants found in our AML patients, a gene prioritization analysis was performed in order to define an *in silico* model based on the physical interaction, regulation, functionality and cell pathway alterations caused by each mutation. The analysis included the interactome of the 28 candidate genes selected by harboring recurrent mutations and displaying SIFT values in the range of the deleteriousness, with significant intrinsic mutation rates (*NPM1*, *DNMT3A*, *NRAS*, *PTPN11*, *IDH2*, *KRAS*, *WT1*, *IDH1*, *RUNX1*, *U2AF1*, *CEBPA*, *TP53*, *CHD4*, *PCDHA6*, *GATA2*, *ASXL1*, *DLG1*, *BCOR*, *PKD1L2*, *SIPA1L2*, *MAGI1*, *FAM70B*, *FCGBP*, *TET2*, *DNAH9*, *TEKT4*, *FLT3*). We identified a network in which 19 out of the 28 genes were found to be significantly more connected that the random expectation (*P =* 0.02) (Fig 3). Moreover, 13 genes (*NPM1*, *DNMT3A*, *NRAS*, *PTPN11*, *IDH2*, *KRAS*, *WT1*, *IDH1*, *RUNX1*, *U2AF1*, *CEBPA*, *TP53*, *CHD4*) also displayed an accumulation of mutations significantly higher that the corresponding healthy controls taken from the 1000 genomes repository (*P*≤0.01) [17]. Focusing on these genes, we found an average of 2 altered genes by patient. Although not always the same pathway was altered, the DNA methylation pathway prevailed above the others. Indeed, more than half of the patients had alterations in the genes involved in this pathway. Moreover, the co-occurrence of mutations in genes involved in less frequent pathways (i.e. signalling activation, chromatin modification, transcription factors and cohesine complex) may trigger the leukemogenesis (Fig 4).

**Fig 5:**
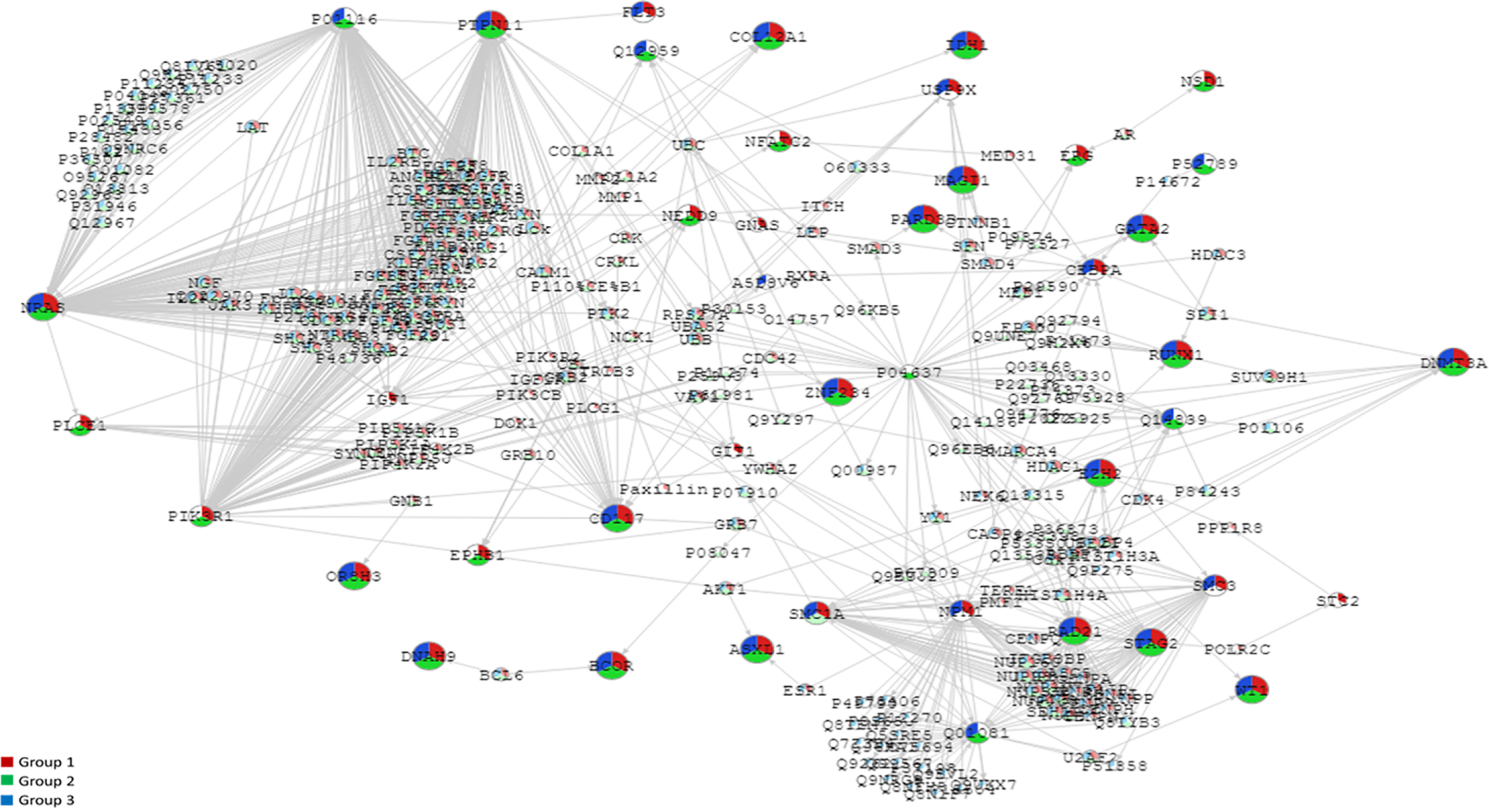
Network-based analysis (SNOW; Babelomics) applied to 28 selected genes. The network was complemented with the co-occurrence relationships, in order to summarize the two kind of significant results. Significant network-based analysis genes are coloured depending their categorical group: patients with NK and/or well- known gene mutations (Group 1, red), patients with NK and without mutations in *NPM1*, *CEBPA* and *FLT3*–ITD (Group 2, green) and IR patients with cytogenetic abnormalities and/or known molecular features (Group 3, blue). Grey edges represent protein-protein interaction, relationships, broad orange dashed lines describe significant co-occurrences.

In addition, according to the *in silico* model, we were able to classify patients in 3 different sets: patients with NK and/or well- known gene mutations that is, *NPM1*, *CEBPA*, and/or *FLT3*–ITD (Group 1, n = 56); patients with NK and lacking mutations in *NPM1*, *CEBPA*, and *FLT3*–ITD (Group 2, n = 22); and IR patients with cytogenetic abnormalities and/or known molecular features, *NPM1*, *CEBPA*, and/or *FLT3*–ITD (Group 3, n = 22). According to these, we found 3 different pathways rather similar between them. Comparing all the pathways, we defined a single *in silico* model including the alterations detected in our sets of patients (Fig 5). Focusing on group 1 and 2, we established a set of seed-genes that might be potentially involved in leukemogenesis due to not being present in patients harboring cytogenetic abnormalities (Group 3), such as *NSD1* (12%), *PLCE1* (9%), *NFATC2* (5%), *EPHB1* (5%), *ERG* (4%), *NEDD9* (3%) and *PIK3R1* (3%). Pathways affected by the presence of these mutations might have a similar effect in the cell than IR-cytogenetic alterations.

### Clinical correlations

Variables included in the regression analyses were age, gender, WBC count, platelet count, percentage of bone marrow and peripheral blood blasts, cytogenetic risk group, MRC code, ECOG, mutational status of *NPM1* (exon 12), *FLT3*, *CEBPA*, and all those genes analyzed by NGS. The follow-up of the patients was updated on December 2017, and all follow-up data were censored at that time point. The median follow-up of surviving patients was 52 months (range, 26 to 164).

No significant correlation was found between the mutations detected and the clinical or biological features studied. In addition, our results did not show statistically significant pattern of cooperating mutations in the studied group of genes. However, results from the elastic net cox regression regarding overall survival, displayed a complex pattern of associations between the different mutations and risk of death, with 26 different variables and age interacting. Hazard ratios are presented in Table 1 and partial dependence plots for the different variables are depicted in S1 Fig. With reference to event-free survival values, a higher risk of adverse event was associated to mutations in *DNAH8*, *TET2*, *RUNX1*, and *WT1*; whilst a lower risk of adverse event was correlated to the presence of mutations in *KDM6A*. Hazard ratios are presented in Table 2. To facilitate the interpretation of these results, partial dependence plots for the different variables are provided in S2 Fig.

**Table 1:** Coefficients and their corresponding Hazard Ratios for overall survival based on predictive factors in 100 *de novo* AML samples.

Variable	Coefficient	HR
Age	0.017	1.018
*FLT3*-ITD	0.150	1.162
*KRAS*	0.009	1.010
*PTPN11*	0.001	1.001
*DNAH8*	0.093	1.097
*IGF1*	-0.026	0.975
*EZH2*	0.263	1.301
*SMC3*	-0.009	0.991
*RAD21*	0.386	1.471
*DNMT3A*	0.015	1.015
*IDH2*	0.047	1.048
*TET2*	0.179	1.196
*SIPA1L2*	-0.027	0.974
*GPR6*	0.114	1.121
*PKD1L2*	0.187	1.205
*RUNX1*	0.060	1.062
*NPM1*	-0.134	0.874
*KCNU1*	-0.114	0.892
*FAM69B*	0.038	1.038
*NLRP5*	-0.164	0.848
*PCDHA7*	0.511	1.667
*ZCCHC1*	-0.025	0.975
*CCNL2*	-0.014	0.986
*TP53*	0.376	1.457
*WT1*	0.165	1.179
*PIK3R*	-0.123	0.884
*OTUD7A*	0.301	1.352

**Table 2:** Hazard ratios for event free survival based on predictive factors in 100 *de novo* AML samples.

Variable	Coefficient	HR
*DNAH8*	0,045	1,046
*KDM6A*	-0,005	0,995
*TET2*	0,057	1,059
*RUNX1*	0,008	1,008
*WT1*	0,147	1,158

## Discussion

This study shows a comprehensive analysis of AML, combining WES with the assessment of somatic mutations by a custom next-generation sequencing panel of targeted genes with network enrichment analysis. In the “discovery cohort”, we identified and validated 64 deleterious somatic variants in 55 genes that were further targeted sequencing in the “validation cohort” of patients along with 32 extra genes previously reported to play a significant role in AML pathogenesis [10]. After bioinformatic filtering of variants, we identified likely deleterious mutations in 73 genes, where 35 were previously reported as recurrent mutated in AML [10, 11]. Network enrichment analysis identified 13 recurrently mutated genes involved in AML-IR pathogenesis. Among them, we defined a single *in silico* model and established a set of seed-genes that may trigger leukemogenesis.

To identify leukemia-specific somatic alterations that could cooperate in the development and progression of leukemia, WES analysis was performed in 7 tumor-normal matched samples. As a normal paired sample, we examined DNA from saliva or from bone marrow at complete remission of each single case (defined according to the recommendations of Cheson *et al*.). To prevent false-positive calls in WES, all stringent filtered variants were resequenced by means of target NGS in paired samples of each patient using a different NGS platform. Among them, 64 deleterious variants were confirmed to be true. Although our protocol took into account several sequencing artifacts as strand bias and other measurable effects derived from poorly mapped reads, some of the not validated mutations could be false positives inherent in the technology used. Furthermore, the issue to confidently identifying indels or the presence of low frequency alleles could explain some false negative calls at the validation resequencing. As a whole, these technical limitations might imply an underestimation of candidate mutated genes in the “discovery cohort”.

In a first step, we identified 55 candidate genes, where 26 were previously reported as mutated in AML patients in previous studies [10]. The identification of previously reported driver mutations validated the robustness of our approach. Then, we extended the analyses through the examination of our candidate genes in an independent validation cohort of 100 *de novo* AML-IR patients. For this propose, a custom panel of 87 genes was designed. Although custom panels require a previous design stage and are optimized by the laboratory, their main advantage is the flexibility they offer, since you can include the regions of your interest and change this design over time (add or remove regions) depending on the needs of each pathology. As no germline matched sample was available from each patient, we implemented a germinality test previously described by our group [14]. In order to filter out the majority of germline variants, we compared the mutations found in our patients against the healthy 1000G cohort and other public variant population databases. Finally, we found a mean of 4.89 mutations per sample (range 0–10) affecting 73 different genes of which 35 genes were recurrently mutated in more than one patient. Recurrent hotspots in genes such as *DNMT3A*, *NRAS*, *KRAS* and *IDH1/2* were also present in several samples. In our whole validation cohort, the most frequent mutated genes were *NPM1*, *DNMT3A*, *FLT3*, *TET2*, and *ASXL1*. These results agreed with those previously reported [10, 11]. When we focussed our analysis on those patients from the validation cohort with same molecular features than the discovery cohort (n = 24), we found somatic mutations with deleterious effect in 100% of patients, mainly in *ASXL1*, *DNMT3A* and/or *RUNX1*. There were other mutated genes within this set of patients such as *DNAH9*, *IDH2*, *WT1*, *DNAH8*, *NRAS* or *TET2*. However, we found that almost 25% of the patients carried *DNMT3A* mutations as an only alteration whilst 13% presented *ASXL1* and *RUNX1* in a simultaneous manner. Although this association was established evaluating a subset of 24 patients, such observations might suggest that the alterations harboured by this set of patients could derive in an aberrant epigenetics regulation. In line with recent reports, highlighting the complex nature of genomic aberrations in leukemia [4, 6, 7], probably the mechanisms that trigger the leukemogenic process could have a common consequence. Functional validation studies will be required to assess the importance of these mutations for AML pathogenesis when well established mutations are not present.

Likewise, exploratory *in silico* approaches were used to provide a reliable approach to prioritize genes that would undergo additional analysis. Candidate genes were selected according to the recurrence of mutations and their SIFT values in the range of the deleteriousness as it has been previously reported [14]. Network analysis resulted in a significantly connected subnetwork in which 19 of 28 genes were significantly more connected that the random expectation. Amidst, 13 genes (*NPM1*, *DNMT3A*, *NRAS*, *PTPN11*, *IDH2*, *KRAS*, *WT1*, *IDH1*, *RUNX1*, *U2AF1*, *CEBPA*, *TP53*, *CHD4*) also displayed an accumulation of mutations significantly higher that the corresponding healthy controls taken from the 1000 genomes repository. This result strongly suggests that all these genes are close in the interactome, as frequently occurs with genes of the same disease [18,19]. The epigenetic regulation is affected in these patients directly or through the interactome neighborhood by specific combinations of mutations that contribute to the arising and maintenance of AML. When we classified patients in 3 different sets according to their harbored mutations [Group 1, (n = 56), Group 2, (n = 22), Group 3, (n = 22)] we established a set of seed-genes that could be potentially involved in leukemogenesis due to not being present in patients with cytogenetic abnormalities (*ERG*, *NSD1*, *PLCE1*, *NFATC2*, *NEDD9*, *PIK3R1*, and *EPHB1*). The consequence of these mutations might have a similar effect in the cell than those presents in IR-cytogenetic alterations. As a member of the ETS (erythroblast transformation-specific) family of transcription factors, *ERG* (ETS-related gene) regulates transcription probably by modifying the structure of the chromatin. In leukemia, *ERG* targets transcription factors such as *GATA2*, which is an important regulator of hematopoietic stem cell and megakaryocyte development and, *RUNX1*, which is involved in the development of normal hematopoiesis. Functional analyses conducted in mice and human CD34 normal and leukemic cells have demonstrated the role of *ERG* in the induction of early myeloid progenitors in leukemia stem cell through the activation of RAS pathway and Pim1 [20–24]. Several studies have established the prognosis impact of *ERG* expression on adult patients with AML, improving the molecular risk stratification of NK-AML [22, 25–29]. Although mutations in *ERG* have not been reported in previous AML studies, 222 different mutations affecting 211 patients in other cancers (n = 22) such as breast, brain or soft tissue have been described. NSD proteins are epigenetic regulators that methylate lysine side chains affecting chromatin organization. This protein is encoded by *NSD1* gene and plays a pivotal role in childhood acute myeloid leukemia (AML). This gene has been described in a cryptic rearrangement with *NUP98* in 15–20% of children with NK-AML. However, the oncogenic fusion transcript is rarely found in adults (~2%) [30–32]. In previous results, mutations in this histone methyltransferase has been detected at low frequency in AML (~3%) and its impact on the outcome of AML patients still remains unclear [10, 33, 34]. *PLCE1* belongs to a phospholipase family that controls gene expression, cell growth and differentiation. A total of 665 different mutations have been reported in several types of cancer. In particular, mutations in *PLCE1* have been described in ~3% of patients with AML [10]. *NFATC2* is a member of a protein family that acts as a transcription factor in immune response participating in the maturation of peripheral lymphocytes [35–38]. Mutations (n = 326) in this gene have also been reported in 293 patients from 19 different types of cancer [10]. Particularly, several studies performed in ALL patients (n = 3308) have associated *NFATC2* mutations with a poor outcome by increasing the risk of asparaginase hypersensitivity [39]. *NEDD9* regulates tyrosine-kinase-based signaling complexes involved in multiple actions such as cell adhesion, migration and apoptosis, affecting cancer metastasis [40–43]. In addition, this gen has been related to BCR receptor in B- and T-cell and the inhibition of migration and dissemination of neoplastic myeloid cells [44–47]. Recently, Pallarès et al. have studied two independent cohorts of AML patients (n = 279) and established *NEDD9* gene expression as a favorable prognosis factor in IR-AML patients subgroup [48]. Moreover, mutations in this gene have been associated with drug resistance in several solid tumors [10]. Gain-of-function mutations in *PIK3R1* have been associated with an oncogenic activation of PIK signaling pathway which plays an important role in the regulation of FGFR family, *PDGFRA*, *PDGFRB* and *KIT* genes, among others [49, 50]. Previous reports have described mutations in *PIK3R1* in leukemia (6.4%), establishing *PIK3R1* as an actionable gen in AML [51, 52]. Finally, human and murine myeloid assays have shown a reduction of BCR-ABL transformation by inhibiting PI3K/AKT pathway [53–56]. Finally, *EPHB1* is a receptor tyrosine kinase involved in normal hematopoietic development and in leukemogenesis [57]. Mutations causing a loss-of-function in this gene have been associated with an aggressive cancer phenotype [58, 59]. In AML, *EPHB1* has been defined as a tumor suppressor that regulates DNA damage response system; therefore, mutations affecting this gene have been correlated with poor overall survival [60, 61]. Moreover, mutations disturbing *EPHB1* methylation have been related with ALL pathogenesis [62]. Since mutations in these seed genes are very rare in previously published AML cohorts [10], they could trigger leukemogenesis only in this subset of AML patients which lack cytogenetic alterations and/or well-known molecular features that is, wild-type *NPM1*, *CEBPA*, and *FLT3*–ITD. For these reason, the impairment of a specific alteration could not be as essential as the interaction of several mutated genes belonging to functionally related categories in the development of AML. Further analysis will focus on the characterization larger and independent cohorts of patients with NK-AML to establish the frequency and their impact on the leukemogenesis. In addition, to improve our knowledge about their interactions, *in vitro* assays will be performed by gene editing technologies. With this purpose, observed mutations will be introduced in cell cultures to transform these results into data of clinical relevance that will be virtually used in the prognostic and therapeutic decision process.

Three major limitations to our study are the relative small sample size, their selection bias and normal-matched samples. We have analyzed an initial cohort of 7 patients by WES and 100 patients by a targeted gene-panel using NGS. As a consequence, to perform a study of a specific group of patients, there were not randomly included in our study. For this reason, it has been difficult to set up a significant independent association for any gene mutation using univariate or multivariate analysis. However, by conducting an elastic net cox regression, we found a complex pattern of associations between the different mutations and risk of death, consistent with previous reports [10–12]. In this regard, we used regularized regression methods in our statistical analyses to reduce over-fitting in small samples and deal appropriately with analyses were the number of variables is high relative to the number of observations [63, 64]. Therefore, more solid evidence based on larger series with long-term follow-up is needed to correlate our results with other clinical data and to clarify the prognostic impact of each mutation. In addition, the use of bone marrow sample from complete remission as “normal” matched control could have led us to discard somatic variants persistent at complete remission. The same for the saliva sample, which could be contaminated with leucocytes. Therefore, we could underestimate candidate mutated genes in our discovery cohort. However, we were interested in those mutations that trigger leukemogenesis, which should not be present when patients achieve complete remission.

In summary, this report describes a complete analysis of AML patients with NK combining WES, targeted NGS and network enrichment approaches. As a result, in 100 AML patients we have described mutations in 73 genes, 35 of them recurrently mutated. Additionally, we have defined a functional module in the interactome of AML pointed 13 recurrent mutated genes significantly involved in the pathogenesis of AML. After classify patients according to their mutations, we defined a single *in silico* model and established a set of seed-genes that may trigger leukemogenesis. These results led us to hypothesize that the perturbation caused in biological key functions as a consequence of gene mutations might be at least as important as the combination of mutations harbored in each patient. Finally, it is clear that the understanding of the events occurred at the clonal hematopoiesis in this disease is still limited and the future potential of discovery of new pathways interactions is high.

## Supporting information

S1 File Extended experimental procedures. (PDF)

S1 Table Clinical characteristics of patients analyzed by WES. (PDF)

S2 Table Mapping results of the discovery cohort from sequencing data. (PDF)

S3 Table All mutations detected in the study cohort using genome GRCh37/hg19 as a reference. (PDF)

S4 Table Clinical data of patients from the validation cohort. (PDF)

S5 Table Mutated genes detected by target-resequencing at the validation cohort. (PDF)

S1 Fig Partial dependence plots for overall survival from different variables. (PDF)

S2 Fig Partial dependence plots for event free survival from the different variables. (PDF)
