# Supplementary material for: The modular network structure of the mutational landscape of Acute Myeloid Leukemia
Source: PLoS One. 2018 Oct 10;13(10):e0202926. doi: 10.1371/journal.pone.0202926 (PMC6179200; doi:10.1371/journal.pone.0202926)
Supplement: S1 File — (PDF) [file pone.0202926.s001.pdf]

The modular network structure of the mutational landscape of Acute Myeloid Leukemia

Mariam Ibáñez,[1,2,3] ¶, José Carbonell-Caballero[4] ¶, Esperanza Such,[1,2] Luz García-Alonso, [5] Alessandro Liquori, [1,2] María López-Pavía[1] , Marta LLop [2,6], Carmen Alonso, [7], Eva Barragán, [2,6] Inés Gomez-Seguí [1,2], Alexander Neef [1], David Hervás [8], Pau Montesinos, [1,2], Guillermo Sanz [1,2], Miguel Angel Sanz, .[1,2], Joaquín Dopazo, [,9,10, 11,*], José Cervera.[1,2,12,*]

[1]Hematology Service, Hospital Universitario y Politécnico La Fe, Valencia, Spain; [2] Centro de Investigacion Biomédica en Red de Cáncer (CIBERONC), Instituto Carlos III, Madrid, Spain; [3]Departamento de Ciencias Biomédicas. Facultad de Ciencias de la Salud. Universidad CEU Cardenal Herrera. Valencia, Spain; [4]ProCURE. Catalan Institute of Oncology. Bellvitge Institute for Biomedical Research (IDIBELL). L'Hospitalet del Llobregat. 08908 Barcelona. Spain; [5]European Molecular Biology Laboratory - European Bioinformatics Institute, Wellcome Genome Campus, CB10 1SD Cambridge, UK; [6]Department of Medical Pathology, Hospital Universitario La Fe, Valencia, Spain; [7]Hematology Service, Hospital Arnau de Villanoba, Valencia, Spain; [8]Biostatistics Unit, IIS La Fe Valencia, Spain; [9]Functional Genomics Node, Spanish National Institute of Bioinformatics at CIPF, Valencia, Spain; [10]Bioinformatics of Rare Diseases (BIER), CIBER de Enfermedades Raras (CIBERER), Valencia, Spain; [11] Clinical Bioinformatics Area. Fundación Progreso y Salud (FPS). CDCA, Hospital Virgen del Rocio. 41013. Sevilla. Spain; [12]Genetics Unit, Hospital Universitario y Politécnico La Fe, Valencia, Spain.

Running title:

The modular network of Acute Myeloid Leukemia

Corresponding author:

José Vicente Cervera Zamora. Hospital Universitario La Fe. Avd. Fernando Abril Martorell, 106. 46026 Valencia, Spain. Phone: +34 961244688; E-mail: cervera_jos@gva.es

Joaquín Dopazo. Clinical Bioinformatics Area. Fundación Progreso y Salud (FPS). CDCA, Hospital Virgen del Rocio. 41013. Sevilla. Spain. E-mail: joaquin.dopazo@gmail.com

Supporting information

S1 File. Extended Experimental Procedures.

S1 File. Extended Experimental Procedures.

Patients

Patients with *de novo* AML with intermediate cytigenetic risk were included in this project. The only limiting criterion for including patients in our study was the availability of the DNA sample for each given time. In all patients, routine test were performed, such as conventional cytogenetics, FISH and NGS, as well as tests for the detection of *FLT3*-ITD and D835 mutations as previously described [14]. Every patient was enrolled in consecutive PETHEMA trials. On December 2017 was performed the last update on clinical data, treatment outcome and follow-up, which was collected prospectively. In accordance with the Declaration of Helsinki, this study was approved by the Research Ethics Board of our hospital (No.2012/0175).

Whole-exome sequencing (WES) was done on matched samples from 7 *de novo* AML patients under the age of 60 years who lacked cytogenetically abnormalities and well known molecular features (*NPM1, CEBPA* and *FLT3*–ITD), as our “discovery cohort” (S1 Table). For each patient, we obtained DNA sample from bone marrow aspiration at diagnosis time. A paired DNA sample from saliva or from bone marrow at complete molecular remission was taken from each patient. Available matched DNA samples was the only limiting criterion. In all cases, Biobank La Fe provided DNA samples.

Additionally, 100 AML intermediate-risk (IR) category patients were selected for the extended analysis as “validation cohort” (S4 Table). Among them, 77% did not harbor cytogenetic abnormalities (n = 77), being 31% with the same molecular characteristics than the discovery cohort (n = 24). All samples were provided by the Biobank La Fe.

DNA extraction and molecular analysis were performed as previously was described by Ibáñez M. et al. [14].

Construction of DNA libraries and sequencing data analysis

In a NextSeq Illumina platform, WES was performed following the manufacturer recommendations. To aim the identification of acquired deleterious variations at coding sequence, WES data was analyzed using a bioinformatics pipeline designed in-house by our group [14]. With this purpose, data from each paired sample was compered to detect somatic variations.

Validation of mutations from candidate genes

Data obtained from our WES analyses was validated by targeted sequencing using an Ion AmpliSeq™ analysed in an Ion Proton™ System as recommended by the manufacturer. This assay allowed us confirms point and indels variations to establish candidate genes. Sequencing data was analyzed using and in-house bioinformatics pipeline as it has been described previously [14].

Targeted resequencing using Haloplex technology and sequence analysis

The Haloplex target enrichment system (Agilent) was performed on an extended cohort (n=100) of patients. We designed a custom panel of 87 genes (S5 Table) using SureDesign Tool (Agilent) for next-generation sequencing, according to the manufacturer's instructions. Fifty five genes were selected from in-house results and 32 genes from previously published AML series [10]. The analyzed genes were: *ACTN2, ASXL1, ATP6AP1, BCOR, BTNL3, C12orf51, CCNL2, CD22, CEBPA, CHD4, CHI3L2, CIC, COL12A1, CSMD1 , DGCR2, DLG1, DNAH8, DNAH9, DNMT3A, EPHB1, EPX, ERG, EZH2, FAM5C, FAM69B, FAM70B, FCGBP, FLT3, GATA2, GIT1, GNAS, GPR6, GRID1, HK2, HNRNPK, HYDIN, IDH1, IDH2, IGF1, INTS12, KCNU1, KDM6A, KIT, KRAS, MAGI1, NRAS, NEDD9, NFATC2, NLRP5, NPM1, NSD1, OR8H3, OTUD7A, PARD3B, PCDHA7, PCMTD1, PHF6, PIK3R1, PKD1L2, PLA1A, PLCE1, PTGIS, PTPN11, RAD21, RUNX1, SIPA1L2, SMC1A, SMC3, STAG2, STC2, SUV39H1, TEKT4, TET2, TP53, TRIM67, TSPAN5, U2AF1, USP34, USP9X, VPS37C, WT1, ZCCHC14, ZCCHC16, ZMYND17, ZNF234, ZNF253* and *ZSWIM1.* Selected variants were annotated as described in Ibáñez M et al. PlosOne 2016 [14].

Next-generation sequencing data from other AML series

We compared our results with AML cases as our discovery cohort from The Cancer Genome Atlas Network (TCGA) [10].

Network analysis

Network analysis of the candidate gene products was carried out as it has been previously reported by our group [14].

Statistical methods

Data were summarized using mean (standard deviation) and median (1st and 3rd quartile) in the case of continuous variables and with relative and absolute frequencies in the case of

categorical variables. Unadjusted time-to-event analysis were performed using the Kaplan-Meier estimate and for comparisons, long-rank tests. All P values reported are two-sided. Multivariable time-to-event analyses were performed using elastic net penalized cox regression models. For the elastic net analyses, an initial alpha value of 0.1 was selected and 500 replicates of 10-fold cross validation were used to estimate the penalization parameter following the one-standard-error rule. Alternative alpha values were used to assess the stability and robustness of the estimates. All statistical analyses were performed using R (version 3.3.3) and the R-package glmnet (version 2.0-5).
