# Supplementary material for: The modular network structure of the mutational landscape of Acute Myeloid Leukemia
Source: PLoS One. 2018 Oct 10;13(10):e0202926. doi: 10.1371/journal.pone.0202926 (PMC6179200; doi:10.1371/journal.pone.0202926)
Supplement: S1 Table — (PDF) [file pone.0202926.s002.pdf]

Supplementary Table S1. Clinical characteristics of patients analyzed by WES.

Patient	#AML 1	#AML 2	#AML 3	#AML 4	#AML 5	#AML 6	#AML 7
Age at Dg	28	60	55	21	20	28	53
Sex	Male	Male	Female	Female	Male	Male	Female
WGS consent	yes	yes	yes	yes	yes	yes	yes
Hemoglobin	9,10	9,30	7,60	9,10	13,40	10,50	10,5
Leucocyte	2,90	2,60	0,80	6,00	3,20	2,10	1,6
Platelets	160	14	157	40	184	182	87
%BM Blast	71	53	14	94	76	79	57
Clinical Cytogenetics	46,XY	46,XY	46,XX	46,XX	46,XY	46,XY	46,XX
FISH Cytocell*	Normal	Normal	Normal	Normal	Normal	Normal	Normal
Relapse	no	no	no	no	no	no	yes
Expired	no	no	yes	no	yes	no	yes
OS (mo)	103	87	9	127	18	147	21
