# Supplementary material for: The modular network structure of the mutational landscape of Acute Myeloid Leukemia
Source: PLoS One. 2018 Oct 10;13(10):e0202926. doi: 10.1371/journal.pone.0202926 (PMC6179200; doi:10.1371/journal.pone.0202926)
Supplement: S2 Table — (PDF) [file pone.0202926.s003.pdf]

Supplementary Table S2. Mapping results from sequencing data.

Patient	Sample	Bases	>Q20	Reads	Mean Read Length (pb)
#AML 1	Tumor	4.553.343	3.986.255	30.976	146
#AML 1	Normal	4.982.533	4.387.849	33.507	148
#AML 2	Tumor	3.695.240	3.253.919	28.213	130
#AML 2	Normal	4.768.382	4.212.709	34.401	138
#AML 3	Tumor	5.064.510	4.438.856	34.046	148
#AML 3	Normal	4.558.711	4.015.547	33.518	136
#AML 4	Tumor	5.455.915	4.781.959	37.135	146
#AML 4	Normal	4.631.925	4.071.348	31.100	148
#AML 5	Tumor	4.849.858	4.280.860	32.608	148
#AML 5	Normal	4.910.242	4.347.146	34.938	140
#AML 6	Tumor	5.319.895	4.707.803	35.834	148
#AML 6	Normal	4.667.911	4.092.747	32.154	145
#AML 7	Tumor	4.997.126	4.409.818	32.786	152
#AML 7	Normal	4.424.748	3.906.619	29.993	147
