# Supplementary material for: The modular network structure of the mutational landscape of Acute Myeloid Leukemia
Source: PLoS One. 2018 Oct 10;13(10):e0202926. doi: 10.1371/journal.pone.0202926 (PMC6179200; doi:10.1371/journal.pone.0202926)
Supplement: S3 Table — (PDF) [file pone.0202926.s004.pdf]

Supplementary Tabala S3. All mutations detected in the study cohort using genome GRCh37/hg19 as a reference.

Patient ID	Sample	Chrom	Start Position	End Position	Gene ID*	Type	Zygosity	Genotype	Ref	Variant	Coverage	Ref Cov	Var Cov	*Gen previously reported at TCG
#AML 1	Tumor	chr1	111783982	111783982	*CHI3L2*	SNP	Het	C/A	C	A	219	110	109	No
#AML 1	Normal	chr1	111783982	111783982	*CHI3L2*	SNP	Het	C/A	C	A	245	131	114	No
#AML 1	Tumor	chr1	111783996	111783996	*CHI3L2*	SNP	Hom	G/G	A	G	221	0	221	No
#AML 1	Normal	chr1	111783996	111783996	*CHI3L2*	SNP	Hom	G/G	A	G	251	0	251	No
#AML 1	Tumor	chr12	112696301	112696301	*IGF1*	SNP	Het	T/C	T	C	267	177	90	No
#AML 1	Tumor	chr16	87445363	87445363	*ZCCHC14*	SNP	Hom	A/A	G	A	176	2	174	No
#AML 1	Normal	chr16	87445363	87445363	*ZCCHC14*	SNP	Hom	A/A	G	A	174	2	172	No
#AML 1	Tumor	chr19	20003109	20003109	*ZNF253*	SNP	Het	A/G	A	G	185	84	101	No
#AML 1	Normal	chr19	20003109	20003109	*ZNF253*	SNP	Het	A/G	A	G	196	99	97	No
#AML 1	Tumor	chr20	50092027	50092027	*NFATC2*	SNP	Hom	G/G	T	G	200	0	200	No
#AML 1	Normal	chr20	50092027	50092027	*NFATC2*	SNP	Hom	G/G	T	G	188	0	188	No
#AML 1	Tumor	chr21	39817525	39817525	*ERG*	SNP	Het	C/T	C	T	444	272	172	No
#AML 1	Tumor	chr3	4185026	4185026	*SUMF1*	SNP	Het	G/A	G	A	252	138	114	No
#AML 1	Normal	chr3	4185026	4185026	*SUMF1*	SNP	Het	G/A	G	A	237	44	193	No
#AML 1	Tumor	chr5	67588982	67588982	*PIK3R1*	SNP	Het	G/T	G	T	294	195	99	No
#AML 1	Normal	chr5	140229845	140229845	*PCDHA7*	SNP	Het	G/A	G	A	45	43	2	No
#AML 1	Tumor	chr8	52733050	52733050	*PCMTD1*	SNP	Hom	A/A	T	A	121	2	119	No
#AML 1	Normal	chr8	52733050	52733050	*PCMTD1*	SNP	Hom	A/A	T	A	160	0	160	No
#AML 1	Tumor	chr9	139617578	139617578	*FAM69B*	SNP	Het	G/A	G	A	63	61	2	No
#AML 1	Tumor	chr9	139617626	139617626	*FAM69B*	SNP	Het	G/A	G	A	62	26	36	No
#AML 1	Normal	chr9	139617626	139617626	*FAM69B*	SNP	Het	G/A	G	A	63	53	10	No
#AML 1	Tumor	chr9	139617634	139617634	*FAM69B*	SNP	Het	C/T	C	T	61	42	19	No
#AML 1	Tumor	chr9	139617662	139617662	*FAM69B*	SNP	Hom	C/C	T	C	36	0	36	No
#AML 1	Normal	chr9	139617662	139617662	*FAM69B*	SNP	Het	T/C	T	C	63	49	14	No
#AML 1	Normal	chrX	48559098	48559098	*SUV39H1*	SNP	Het	T/G	T	G	52	50	2	No
#AML 1	Tumor	chrX	153660206	153660206	*ATP6AP1*	SNP	Het	G/C	G	C	137	29	108	No
#AML 2	Tumor	chr1	111783996	111783996	*CHI3L2*	SNP	Hom	G/G	A	G	258	0	258	No
#AML 2	Normal	chr1	111783996	111783996	*CHI3L2*	SNP	Hom	G/G	A	G	340	0	340	No
#AML 2	Tumor	chr11	55890103	55890103	*OR8H3*	SNP	Het	C/T	C	T	26	15	11	No
#AML 2	Normal	chr11	55890103	55890103	*OR8H3*	SNP	Het	C/T	C	T	67	52	15	No
#AML 2	Normal	chr11	60906279	60906279	*VPS37C*	SNP	Het	G/T	G	T	267	204	63	No
#AML 2	Tumor	chr11	60906320	60906320	*VPS37C*	SNP	Het	T/C	T	C	90	39	51	No
#AML 2	Normal	chr11	60906320	60906320	*VPS37C*	SNP	Het	T/C	T	C	261	198	63	No
#AML 2	Tumor	chr12	11214360	11214360	*PRH1*	SNP	Het	C/A	C	A	205	180	25	No
#AML 2	Normal	chr12	11214360	11214360	*PRH1*	SNP	Het	C/T	C	T	236	136	100	No
#AML 2	Tumor	chr12	11214363	11214363	*PRH1*	SNP	Het	T/C	T	C	207	181	26	No
#AML 2	Normal	chr12	11214363	11214363	*PRH1*	SNP	Het	T/C	T	C	314	269	45	No
#AML 2	Tumor	chr12	11214368	11214368	*PRH1*	SNP	Het	T/C	T	C	225	183	42	No
#AML 2	Normal	chr12	11214368	11214368	*PRH1*	SNP	Het	T/C	T	C	332	271	61	No
#AML 2	Tumor	chr12	11214386	11214386	*PRH1*	SNP	Het	T/G	T	G	223	182	41	No
#AML 2	Normal	chr12	11214386	11214386	*PRH1*	SNP	Het	T/G	T	G	332	270	62	No
#AML 2	Tumor	chr12	11214392	11214392	*PRH1*	SNP	Het	G/A	G	A	224	182	42	No
#AML 2	Normal	chr12	11214392	11214392	*PRH1*	SNP	Het	G/A	G	A	331	269	62	No
#AML 2	Tumor	chr12	11214437	11214437	*PRH1*	SNP	Het	T/C	T	C	223	181	42	No
#AML 2	Normal	chr12	11214437	11214437	*PRH1*	SNP	Het	T/C	T	C	331	268	63	No
#AML 2	Tumor	chr12	11214455	11214455	*PRH1*	SNP	Het	T/C	T	C	224	182	42	No
#AML 2	Normal	chr12	11214455	11214455	*PRH1*	SNP	Het	T/C	T	C	332	270	62	No
#AML 2	Tumor	chr12	11214472	11214472	*PRH1*	SNP	Het	A/G	A	G	220	178	42	No
#AML 2	Normal	chr12	11214472	11214472	*PRH1*	SNP	Het	A/G	A	G	324	259	65	No
#AML 2	Tumor	chr12	11214495	11214495	*PRH1*	SNP	Het	T/C	T	C	201	168	33	No
#AML 2	Normal	chr12	11214495	11214495	*PRH1*	SNP	Het	T/C	T	C	296	238	58	No
#AML 2	Tumor	chr16	50666209	50666209	*NKD1*	SNP	Het	G/A	G	A	167	153	14	No
#AML 2	Tumor	chr16	87445363	87445363	*ZCCHC14*	SNP	Het	G/A	G	A	61	33	28	No
#AML 2	Normal	chr16	87445363	87445363	*ZCCHC14*	SNP	Het	G/A	G	A	74	29	45	No
#AML 2	Tumor	chr16	87445422	87445422	*ZCCHC14*	SNP	Het	T/C	T	C	59	57	2	No
#AML 2	Tumor	chr17	76967674	76967674	*LGALS3BP*	SNP	Het	G/C	G	C	39	26	13	No
#AML 2	Tumor	chr19	20003109	20003109	*ZNF253*	SNP	Het	A/G	A	G	95	47	48	No
#AML 2	Normal	chr19	20003109	20003109	*ZNF253*	SNP	Het	A/G	A	G	190	96	94	No
#AML 2	Tumor	chr20	50092027	50092027	*NFATC2*	SNP	Het	T/G	T	G	86	51	35	No
#AML 2	Normal	chr20	50092027	50092027	*NFATC2*	SNP	Het	T/G	T	G	144	112	32	No
#AML 2	Tumor	chr22	19028735	19028735	*DGCR2*	SNP	Het	G/A	G	A	125	71	54	No
#AML 2	Tumor	chr3	4185026	4185026	*SUMF1*	SNP	Het	G/A	G	A	175	84	91	No
#AML 2	Normal	chr3	4185026	4185026	*SUMF1*	SNP	Het	G/A	G	A	304	146	158	No
#AML 2	Tumor	chr3	119334879	119334879	*PLA1A*	SNP	Het	G/A	G	A	157	103	54	No
#AML 2	Normal	chr8	52733050	52733050	*PCMTD1*	SNP	Het	T/A	T	A	83	46	37	No
#AML 2	Tumor	chr9	139617626	139617626	*FAM69B*	SNP	Het	G/A	G	A	17	8	9	No
#AML 2	Normal	chr9	139617626	139617626	*FAM69B*	SNP	Het	G/A	G	A	44	36	8	No
#AML 2	Tumor	chr9	139617662	139617662	*FAM69B*	SNP	Hom	C/C	T	C	12	0	12	No
#AML 2	Normal	chr9	139617662	139617662	*FAM69B*	SNP	Het	T/C	T	C	42	13	29	No
#AML 3	Tumor	chr1	89449434	89449434	*CCBL2*	SNP	Het	T/C	T	C	394	379	15	No
#AML 3	Tumor	chr1	111783980	111783980	*CHI3L2*	SNP	Het	C/T	C	T	185	114	71	No
#AML 3	Tumor	chr1	111783982	111783982	*CHI3L2*	SNP	Het	C/A	C	A	183	102	81	No
#AML 3	Normal	chr1	111783982	111783982	*CHI3L2*	SNP	Het	C/A	C	A	89	42	47	No
#AML 3	Tumor	chr1	111783996	111783996	*CHI3L2*	SNP	Hom	G/G	A	G	186	2	184	No
#AML 3	Normal	chr1	111783996	111783996	*CHI3L2*	SNP	Hom	G/G	A	G	92	1	91	No
#AML 3	Tumor	chr1	231342500	231342500	*TRIM67*	SNP	Het	C/T	C	T	508	346	162	No
#AML 3	Normal	chr11	55890103	55890103	*OR8H3*	SNP	Het	C/T	C	T	12	7	5	No
#AML 3	Tumor	chr12	11214368	11214368	*PRH1*	SNP	Het	T/C	T	C	199	175	24	No
#AML 3	Normal	chr12	11214368	11214368	*PRH1*	SNP	Het	T/C	T	C	115	98	17	No
#AML 3	Tumor	chr12	11214386	11214386	*PRH1*	SNP	Het	T/G	T	G	202	178	24	No
#AML 3	Normal	chr12	11214386	11214386	*PRH1*	SNP	Het	T/G	T	G	115	98	17	No
#AML 3	Tumor	chr12	11214392	11214392	*PRH1*	SNP	Het	G/A	G	A	202	178	24	No
#AML 3	Normal	chr12	11214392	11214392	*PRH1*	SNP	Het	G/A	G	A	116	99	17	No
#AML 3	Tumor	chr12	11214437	11214437	*PRH1*	SNP	Het	T/C	T	C	197	174	23	No
#AML 3	Normal	chr12	11214437	11214437	*PRH1*	SNP	Het	T/C	T	C	114	98	16	No
#AML 3	Tumor	chr12	11214455	11214455	*PRH1*	SNP	Het	T/C	T	C	190	165	25	No
#AML 3	Normal	chr12	11214455	11214455	*PRH1*	SNP	Het	T/C	T	C	114	97	17	No
#AML 3	Tumor	chr12	11214472	11214472	*PRH1*	SNP	Het	A/G	A	G	184	158	26	No
#AML 3	Normal	chr12	11214472	11214472	*PRH1*	SNP	Het	A/G	A	G	112	95	17	No
#AML 3	Tumor	chr12	11214495	11214495	*PRH1*	SNP	Het	T/C	T	C	168	147	21	No
#AML 3	Normal	chr12	11214495	11214495	*PRH1*	SNP	Het	T/C	T	C	100	88	12	No
#AML 3	Normal	chr15	31851177	31851177	*OTUD7A*	SNP	Het	T/C	T	C	117	113	4	No
#AML 3	Tumor	chr16	87445363	87445363	*ZCCHC14*	SNP	Hom	A/A	G	A	200	3	197	No
#AML 3	Normal	chr16	87445363	87445363	*ZCCHC14*	SNP	Hom	A/A	G	A	149	0	149	No
#AML 3	Tumor	chr19	20003042	20003042	*ZNF253*	SNP	Het	T/C	T	C	165	105	60	No
#AML 3	Tumor	chr19	20003109	20003109	*ZNF253*	SNP	Het	A/G	A	G	159	79	80	No

#AML 3	Normal	chr19	20003109	20003109	*ZNF253*	SNP	Het	A/G	A	G	76	46	30	No
#AML 3	Tumor	chr3	134920337	134920337	*EPHB1*	SNP	Het	G/A	G	A	258	172	86	No
#AML 3	Tumor	chr8	52733050	52733050	*PCMTD1*	SNP	Het	T/A	T	A	142	68	74	No
#AML 3	Normal	chr8	52733050	52733050	*PCMTD1*	SNP	Het	T/A	T	A	117	62	55	No
#AML 3	Tumor	chr9	139617662	139617662	*FAM69B*	SNP	Het	T/C	T	C	65	41	24	No
#AML 3	Normal	chr9	139617662	139617662	*FAM69B*	SNP	Het	T/C	T	C	60	35	25	No
#AML 3	Normal	chr9	139617665	139617665	*FAM69B*	SNP	Het	A/G	A	G	61	59	2	No
#AML 4	Tumor	chr1	111783982	111783982	*CHI3L2*	SNP	Hom	A/A	C	A	283	0	283	No
#AML 4	Normal	chr1	111783982	111783982	*CHI3L2*	SNP	Hom	A/A	C	A	290	0	290	No
#AML 4	Normal	chr1	111783983	111783983	*CHI3L2*	SNP	Het	G/A	G	A	305	291	14	No
#AML 4	Tumor	chr1	111783996	111783996	*CHI3L2*	SNP	Hom	G/G	A	G	295	0	295	No
#AML 4	Normal	chr1	111783996	111783996	*CHI3L2*	SNP	Hom	G/G	A	G	307	0	307	No
#AML 4	Normal	chr11	55890013	55890013	*OR8H3*	SNP	Het	G/A	G	A	42	40	2	No
#AML 4	Tumor	chr11	55890132	55890132	*OR8H3*	SNP	Het	C/T	C	T	21	11	10	No
#AML 4	Tumor	chr12	11214360	11214360	*PRH1*	SNP	Het	C/T	C	T	170	115	55	No
#AML 4	Normal	chr12	11214360	11214360	*PRH1*	SNP	Het	C/T	C	T	212	144	68	No
#AML 4	Tumor	chr12	102811603	102811603	*IGF1*	SNP	Het	C/T	C	T	70	61	9	No
#AML 4	Tumor	chr16	87445363	87445363	*ZCCHC14*	SNP	Hom	A/A	G	A	221	0	221	No
#AML 4	Normal	chr16	87445363	87445363	*ZCCHC14*	SNP	Hom	A/A	G	A	141	0	141	No
#AML 4	Tumor	chr19	20003109	20003109	*ZNF253*	SNP	Het	A/G	A	G	188	85	103	No
#AML 4	Normal	chr19	20003109	20003109	*ZNF253*	SNP	Het	A/G	A	G	270	141	129	No
#AML 4	Tumor	chr19	44660819	44660819	*ZNF234*	SNP	Het	C/T	C	T	335	278	57	No
#AML 4	Tumor	chr3	4185026	4185026	*SUMF1*	SNP	Het	G/A	G	A	308	169	139	No
#AML 4	Normal	chr3	4185026	4185026	*SUMF1*	SNP	Het	G/A	G	A	318	165	153	No
#AML 4	Tumor	chr5	140215305	140215305	*PCDHA7*	SNP	Het	C/T	C	T	214	118	96	No
#AML 4	Tumor	chr8	52733050	52733050	*PCMTD1*	SNP	Hom	A/A	T	A	183	3	180	No
#AML 4	Normal	chr8	52733050	52733050	*PCMTD1*	SNP	Hom	A/A	T	A	119	0	119	No
#AML 4	Normal	chrX	111698613	111698613	*ZCCHC16*	SNP	Het	T/C	T	C	474	225	249	No
#AML 5	Tumor	chr1	111783982	111783982	*CHI3L2*	SNP	Het	C/A	C	A	323	169	154	No
#AML 5	Normal	chr1	111783982	111783982	*CHI3L2*	SNP	Het	C/A	C	A	369	196	173	No
#AML 5	Tumor	chr1	111783996	111783996	*CHI3L2*	SNP	Hom	G/G	A	G	334	0	334	No
#AML 5	Normal	chr11	55890022	55890022	*OR8H3*	SNP	Het	T/C	T	C	46	44	2	No
#AML 5	Tumor	chr12	11214360	11214360	*PRH1*	SNP	Het	C/A	C	A	364	299	65	No
#AML 5	Normal	chr12	11214360	11214360	*PRH1*	SNP	Het	C/A	C	A	433	350	83	No
#AML 5	Tumor	chr12	11214363	11214363	*PRH1*	SNP	Het	T/C	T	C	365	300	65	No
#AML 5	Normal	chr12	11214363	11214363	*PRH1*	SNP	Het	T/C	T	C	433	350	83	No
#AML 5	Tumor	chr12	11214368	11214368	*PRH1*	SNP	Het	T/C	T	C	404	301	103	No
#AML 5	Normal	chr12	11214368	11214368	*PRH1*	SNP	Het	T/C	T	C	462	347	115	No
#AML 5	Tumor	chr12	11214386	11214386	*PRH1*	SNP	Het	T/G	T	G	407	303	104	No
#AML 5	Normal	chr12	11214386	11214386	*PRH1*	SNP	Het	T/G	T	G	462	350	112	No
#AML 5	Tumor	chr12	11214392	11214392	*PRH1*	SNP	Het	G/A	G	A	408	303	105	No
#AML 5	Normal	chr12	11214392	11214392	*PRH1*	SNP	Het	G/A	G	A	465	352	113	No
#AML 5	Tumor	chr12	11214437	11214437	*PRH1*	SNP	Het	T/C	T	C	400	298	102	No
#AML 5	Normal	chr12	11214437	11214437	*PRH1*	SNP	Het	T/C	T	C	455	343	112	No
#AML 5	Tumor	chr12	11214455	11214455	*PRH1*	SNP	Het	T/C	T	C	403	298	105	No
#AML 5	Normal	chr12	11214455	11214455	*PRH1*	SNP	Het	T/C	T	C	453	341	112	No
#AML 5	Tumor	chr12	11214472	11214472	*PRH1*	SNP	Het	A/G	A	G	397	295	102	No
#AML 5	Normal	chr12	11214472	11214472	*PRH1*	SNP	Het	A/G	A	G	443	330	113	No
#AML 5	Tumor	chr12	11214495	11214495	*PRH1*	SNP	Het	T/C	T	C	359	269	90	No
#AML 5	Normal	chr12	11214495	11214495	*PRH1*	SNP	Het	T/C	T	C	391	297	94	No
#AML 5	Tumor	chr16	70843739	70843739	*HYDIN*	SNP	Het	T/G	T	G	202	59	143	No
#AML 5	Normal	chr16	87445352	87445352	*ZCCHC14*	SNP	Het	C/T	C	T	60	58	2	No
#AML 5	Tumor	chr19	44660791	44660791	*ZNF234*	SNP	Het	G/A	G	A	404	176	228	No
#AML 5	Normal	chr19	44660791	44660791	*ZNF234*	SNP	Het	G/A	G	A	498	259	239	No
#AML 5	Tumor	chr20	50092027	50092027	*NFATC2*	SNP	Hom	G/G	T	G	124	0	124	No
#AML 5	Normal	chr20	50092027	50092027	*NFATC2*	SNP	Hom	G/G	T	G	80	0	80	No
#AML 5	Tumor	chr8	52733050	52733050	*PCMTD1*	SNP	Het	T/A	T	A	113	52	61	No
#AML 5	Normal	chr8	52733050	52733050	*PCMTD1*	SNP	Het	T/A	T	A	72	33	39	No
#AML 6	Tumor	chr1	89449399	89449399	*CCBL2*	SNP	Het	T/C	T	C	598	577	21	No
#AML 6	Tumor	chr1	89449434	89449434	*CCBL2*	SNP	Het	T/C	T	C	601	577	24	No
#AML 6	Tumor	chr1	89449483	89449483	*CCBL2*	SNP	Het	C/G	C	G	593	572	21	No
#AML 6	Tumor	chr1	111783982	111783982	*CHI3L2*	SNP	Het	C/A	C	A	357	184	173	No
#AML 6	Normal	chr1	111783982	111783982	*CHI3L2*	SNP	Het	C/A	C	A	343	163	180	No
#AML 6	Tumor	chr1	111783996	111783996	*CHI3L2*	SNP	Hom	G/G	A	G	365	0	365	No
#AML 6	Normal	chr1	111783996	111783996	*CHI3L2*	SNP	Hom	G/G	A	G	354	0	354	No
#AML 6	Tumor	chr11	55890160	55890160	*OR8H3*	SNP	Het	T/C	T	C	61	58	3	No
#AML 6	Tumor	chr12	11214360	11214360	*PRH1*	SNP	Het	C/A	C	A	386	345	41	No
#AML 6	Normal	chr12	11214360	11214360	*PRH1*	SNP	Het	C/A	C	A	303	262	41	No
#AML 6	Tumor	chr12	11214363	11214363	*PRH1*	SNP	Het	T/C	T	C	386	347	39	No
#AML 6	Normal	chr12	11214363	11214363	*PRH1*	SNP	Het	T/C	T	C	304	263	41	No
#AML 6	Tumor	chr12	11214368	11214368	*PRH1*	SNP	Het	T/C	T	C	408	345	63	No
#AML 6	Normal	chr12	11214368	11214368	*PRH1*	SNP	Het	T/C	T	C	322	262	60	No
#AML 6	Tumor	chr12	11214386	11214386	*PRH1*	SNP	Het	T/G	T	G	414	354	60	No
#AML 6	Normal	chr12	11214386	11214386	*PRH1*	SNP	Het	T/G	T	G	330	269	61	No
#AML 6	Tumor	chr12	11214392	11214392	*PRH1*	SNP	Het	G/A	G	A	414	352	62	No
#AML 6	Normal	chr12	11214392	11214392	*PRH1*	SNP	Het	G/A	G	A	332	271	61	No
#AML 6	Tumor	chr12	11214437	11214437	*PRH1*	SNP	Het	T/C	T	C	408	345	63	No
#AML 6	Normal	chr12	11214437	11214437	*PRH1*	SNP	Het	T/C	T	C	328	267	61	No
#AML 6	Tumor	chr12	11214455	11214455	*PRH1*	SNP	Het	T/C	T	C	405	342	63	No
#AML 6	Normal	chr12	11214455	11214455	*PRH1*	SNP	Het	T/C	T	C	325	264	61	No
#AML 6	Tumor	chr12	11214472	11214472	*PRH1*	SNP	Het	A/G	A	G	396	335	61	No
#AML 6	Normal	chr12	11214472	11214472	*PRH1*	SNP	Het	A/G	A	G	315	253	62	No
#AML 6	Tumor	chr12	11214495	11214495	*PRH1*	SNP	Het	T/C	T	C	348	294	54	No
#AML 6	Normal	chr12	11214495	11214495	*PRH1*	SNP	Het	T/C	T	C	275	226	49	No
#AML 6	Normal	chr15	31851193	31851193	*OTUD7A*	SNP	Het	T/C	T	C	339	203	136	No
#AML 6	Tumor	chr16	87445363	87445363	*ZCCHC14*	SNP	Het	G/A	G	A	115	52	63	No
#AML 6	Normal	chr16	87445363	87445363	*ZCCHC14*	SNP	Het	G/A	G	A	136	80	56	No
#AML 6	Normal	chr20	44512300	44512300	*ZSWIM1*	SNP	Het	G/A	G	A	250	164	86	No
#AML 6	Tumor	chr20	50092027	50092027	*NFATC2*	SNP	Het	T/G	T	G	217	112	105	No
#AML 6	Normal	chr20	50092027	50092027	*NFATC2*	SNP	Het	T/G	T	G	138	72	66	No
#AML 6	Tumor	chr3	4185026	4185026	*SUMF1*	SNP	Het	G/A	G	A	281	143	138	No
#AML 6	Normal	chr3	4185026	4185026	*SUMF1*	SNP	Het	G/A	G	A	234	135	99	No
#AML 6	Tumor	chr5	140215301	140215301	*PCDHA7*	SNP	Het	G/A	G	A	44	42	2	No
#AML 6	Normal	chr5	140229827	140229827	*PCDHA7*	SNP	Het	C/T	C	T	55	53	2	No
#AML 6	Tumor	chr8	52733050	52733050	*PCMTD1*	SNP	Hom	A/A	T	A	182	0	182	No
#AML 6	Normal	chr8	52733050	52733050	*PCMTD1*	SNP	Hom	A/A	T	A	108	0	108	No
#AML 6	Tumor	chr9	139617626	139617626	*FAM69B*	SNP	Het	G/A	G	A	55	27	28	No
#AML 6	Normal	chr9	139617626	139617626	*FAM69B*	SNP	Het	G/A	G	A	44	26	18	No
#AML 6	Tumor	chr9	139617662	139617662	*FAM69B*	SNP	Het	T/C	T	C	39	0	39	No
#AML 6	Normal	chr9	139617662	139617662	*FAM69B*	SNP	Hom	C/C	T	C	29	0	29	No

#AML 7	Normal	chr1	89449434	89449434	*CCBL2*	SNP	Het	T/C	T	C	179	171	8	No
#AML 7	Tumor	chr1	111783996	111783996	*CHI3L2*	SNP	Hom	G/G	A	G	375	0	375	No
#AML 7	Normal	chr1	111783996	111783996	*CHI3L2*	SNP	Hom	G/G	A	G	365	0	365	No
#AML 7	Tumor	chr1	236906269	236906269	*ACTN2*	SNP	Het	G/A	G	A	590	523	67	No
#AML 7	Tumor	chr11	55890152	55890152	*OR8H3*	SNP	Het	T/C	T	C	57	55	2	No
#AML 7	Tumor	chr11	60906294	60906294	*VPS37C*	SNP	Het	G/A	G	A	412	353	59	No
#AML 7	Tumor	chr12	11214360	11214360	*PRH1*	SNP	Het	C/T	C	T	254	165	89	No
#AML 7	Normal	chr12	11214360	11214360	*PRH1*	SNP	Het	C/T	C	T	212	133	79	No
#AML 7	Tumor	chr12	11214368	11214368	*PRH1*	SNP	Het	T/C	T	C	316	300	16	No
#AML 7	Tumor	chr12	11214386	11214386	*PRH1*	SNP	Het	T/G	T	G	315	299	16	No
#AML 7	Tumor	chr12	11214392	11214392	*PRH1*	SNP	Het	G/A	G	A	316	300	16	No
#AML 7	Tumor	chr12	11214455	11214455	*PRH1*	SNP	Het	T/C	T	C	314	298	16	No
#AML 7	Tumor	chr12	11214495	11214495	*PRH1*	SNP	Het	T/C	T	C	294	279	15	No
#AML 7	Tumor	chr16	87445411	87445411	*ZCCHC14*	SNP	Het	G/A	G	A	152	77	75	No
#AML 7	Normal	chr16	87445411	87445411	*ZCCHC14*	SNP	Het	G/A	G	A	81	37	44	No
#AML 7	Tumor	chr19	20003109	20003109	*ZNF253*	SNP	Het	A/G	A	G	217	120	97	No
#AML 7	Normal	chr19	20003109	20003109	*ZNF253*	SNP	Het	A/G	A	G	228	113	115	No
#AML 7	Tumor	chr5	140229827	140229827	*PCDHA7*	SNP	Het	C/T	C	T	36	34	2	No
#AML 7	Tumor	chr8	52733050	52733050	*PCMTD1*	SNP	Het	T/A	T	A	130	70	60	No
#AML 7	Normal	chr8	52733050	52733050	*PCMTD1*	SNP	Het	T/A	T	A	77	36	41	No
#AML 7	Tumor	chr9	139617626	139617626	*FAM69B*	SNP	Hom	A/A	G	A	77	0	77	No
#AML 7	Normal	chr9	139617626	139617626	*FAM69B*	SNP	Het	G/A	G	A	56	3	53	No
#AML 7	Tumor	chr9	139617662	139617662	*FAM69B*	SNP	Hom	C/C	T	C	52	0	52	No
#AML 7	Normal	chr9	139617662	139617662	*FAM69B*	SNP	Hom	C/C	T	C	44	0	44	No
#AML 7	Tumor	chrX	48559005	48559005	*SUV39H1*	SNP	Het	A/G	A	G	231	163	68	No

A database
