# Supplementary material for: The modular network structure of the mutational landscape of Acute Myeloid Leukemia
Source: PLoS One. 2018 Oct 10;13(10):e0202926. doi: 10.1371/journal.pone.0202926 (PMC6179200; doi:10.1371/journal.pone.0202926)
Supplement: S4 Table — (PDF) [file pone.0202926.s005.pdf]

**Supplementary Tabala S4. Clinical data of patients from the extended cohort.**

| Characteristics | All patients (N) |
|---|---|
| Overall | 100 |
| Gender | |
| Male | 60 |
| Female | 40 |
| Cytogenetics | |
| Normal Kariotype | 77 |
| Other | 23 |
| FAB subtype | |
| M0 | 9 |
| M1 | 25 |
| M2 | 28 |
| M4 | 24 |
| M5 | 13 |
| M6 | 1 |
| MRC Code | |
| 3 | 77 |
| 4 | 11 |
| 5 | 1 |
| 9 | 2 |
| 10 | 3 |
| 11 | 6 |
| White blood cell count ($\times 10^9$/L) | |
| Median | 23,1 |
| Range | 0,8-324 |
| Platelet count ($\times 10^9$/L) | |
| Median | 61 |
| Range | 7-631 |
| Haemoglobin count ($\times 10^9$/L) | |
| Median | 9,2 |
| Range | 5,60-15,60 |
| Overall survival (mo) | |
| Median | 24 |
| Range | 0-126 |
| Death | 71 |
| CR achievement | 64 |
| Relapse | 21 |
