# Supplementary material for: The modular network structure of the mutational landscape of Acute Myeloid Leukemia
Source: PLoS One. 2018 Oct 10;13(10):e0202926. doi: 10.1371/journal.pone.0202926 (PMC6179200; doi:10.1371/journal.pone.0202926)
Supplement: S5 Table — (PDF) [file pone.0202926.s006.pdf]

Supplementary Tabala S5. Mutated genes detected by target-resequencing at the extended cohort. (Recurrent mutations are label in sky-blue)

Patient ID	Age	Gender	Status	FLT3-ITD	DNMT3A	NPM1	DNAH8	DNAH9	TET2
#AML_EC_12	28	1	Alive	0				p.Gln2998Terv/p.Leu1479Pro	
#AML_EC_25	63	2	Dead	1	p.Arg320Ter	p.Trp288fs			
#AML_EC_29	47	1	Alive	1	p.Arg882Cys	p.Trp288fs			
#AML_EC_64	75	2	Dead	0	p.Asp702Glu/p.Tyr448Ter			p.Gln445Arg	p.Arg1739fs/p.Glu1137fs/p.Ser1738fs
#AML_EC_83	61	1	Dead	0			p.Arg4490Cys	p.Gly4214Cys	p.Gln1527Ter
#AML_EC_4	52	1	Dead	0				p.Ala4Pro	
#AML_EC_56	71	1	Dead	1		p.Trp288fs		p.Arg2805His	p.Glu1162fs/p
#AML_EC_8	30	2	Alive	0				p.Ala4Pro	
#AML_EC_95	65	1	Dead	0		p.Trp288fs	p.Gln3225Lys|		
#AML_EC_49	73	1	Dead	0		p.Trp288fs			
#AML_EC_22	69	1	Dead	0			p.Ala246Val	p.Leu3866Phe	p.Phe854fs/p.
#AML_EC_36	71	2	Alive	0	p.Arg882Cys	p.Trp288fs	p.Ala4350Val		
#AML_EC_62	50	2	Alive	0					
#AML_EC_31	66	1	Dead	0				p.Pro3519Thr	
#AML_EC_11	20	1	Dead	0					
#AML_EC_77	68	1	Dead	0		p.Trp288fs	p.Arg4687Gln		p.Ser153fs
#AML_EC_1	65	1	Dead	0					p.Arg1261Cys
#AML_EC_10	68	1	Dead	1					
#AML_EC_10(	67	2	Dead	1	p.Trp860Arg		p.Gly215Glu/p.Ile2780Thr		
#AML_EC_13	30	2	Alive	0					
#AML_EC_14	45	2	Dead	0					p.Arg1465Ter/p.Met1333Arg
#AML_EC_15	68	1	Dead	1	p.Arg882His				
#AML_EC_16	66	2	Dead	0			p.Leu2696Pro	p.Asp840Gly	
#AML_EC_17	73	2	Dead	1			p.Ala3823Val|	p.Gly4214Cys	
#AML_EC_18	57	1	Alive	0		p.Trp288fs	p.Thr4503Ser		p.Thr1884Ala
#AML_EC_19	58	1	Dead	0					p.Gln591Ter/p.Ser1776fs
#AML_EC_2	54	2	Dead	0			p.Arg1072Gln/p.(	p.Ser237Pro	
#AML_EC_20	63	1	Dead	0					
#AML_EC_21	65	1	Dead	0	p.Leu547Ile/p.Leu547Pro				
#AML_EC_23	30	2	Dead	1		p.Trp288fs			
#AML_EC_24	39	1	Alive	0		p.Trp288fs	p.Arg1422His		
#AML_EC_26	70	2	Dead	0			p.Ile2780Thr		p.Ser152fs
#AML_EC_27	70	1	Dead	0					p.Asn275fs/p.
#AML_EC_28	63	1	Alive	0					p.Glu537fs/p.Gln1068Ter/p.Thr1884Ala
#AML_EC_3	61	1	Dead	0			p.Arg1072Gln/p.(	p.Ser237Pro	
#AML_EC_30	50	2	Dead	1					
#AML_EC_32	74	1	Dead	0			p.Asn2375His		
#AML_EC_33	63	2	Dead	0	p.Gly543Cys	p.Trp288fs		p.Glu2440Lys	
#AML_EC_34	55	2	Dead	1	p.Arg882Cys	p.Trp288fs			
#AML_EC_35	60	2	Dead	0					
#AML_EC_37	57	2	Dead	0	p.Arg882Cys		p.Thr3756Pro		p.Glu537fs
#AML_EC_38	38	1	Dead	0	p.Arg882Cys	p.Trp288fs	p.Thr3755Pro		p.Pro1723Ser/p.Tyr867His
#AML_EC_39	78	1	Dead	0		p.Trp288fs			
#AML_EC_40	41	2	Dead	0			p.Glu1636Asp		
#AML_EC_41	43	1	Dead	0					
#AML_EC_42	67	2	Dead	0			p.Arg4687Gln		
#AML_EC_43	71	1	Dead	0					
#AML_EC_44	63	1	Dead	1					
#AML_EC_45	62	2	Alive	0	p.Arg882His	p.Trp288fs		p.Asn14Thr/p.Glu13Ala/p.Leu130Met	
#AML_EC_46	35	1	Alive	0					
#AML_EC_47	73	2	Dead	1	p.Ala910Pro/p.Arg771Ter		p.Ile2780Thr		
#AML_EC_48	62	1	Dead	1	p.Ala910Pro/p.Arg771Ter		p.Ile2780Thr		
#AML_EC_5	73	1	Dead	0					
#AML_EC_50	17	1	Dead	0	p.Arg792fs/p.Ser714Cys		p.Thr3756Pro|		p.Ile1873Thr
#AML_EC_51	34	2	Alive	0				p.Ala1795Ser	
#AML_EC_52	75	1	Dead	1	p.Arg882His	p.Trp290fs	p.Thr3924Met		p.Leu1899His
#AML_EC_53	61	1	Dead	1					
#AML_EC_54	31	1	Dead	0				p.Asn14Thr/p.Glu13Ala/p.Glu13	
#AML_EC_55	21	2	Alive	1				p.Ala1795Ser	
#AML_EC_57	57	2	Dead	1		p.Trp288fs			
#AML_EC_58	50	2	Dead	0				p.Met846Ile	
#AML_EC_59	61	1	Alive	0	p.Arg882Cys	p.Trp288fs			
#AML_EC_6	81	1	Dead	0	p.Arg882His				
#AML_EC_60	72	2	Dead	0	p.Gly543Cys				
#AML_EC_61	77	1	Dead	NA			p.Arg1463Lys		p.Ser1776fs
#AML_EC_63	39	1	Dead	1	p.Arg882Ser				
#AML_EC_65	88	1	Dead	0	p.Val622fs	p.Trp288fs		p.Gln445Arg	
#AML_EC_66	35	1	Alive	0					
#AML_EC_67	77	2	Dead	1		p.Trp288fs			p.Arg1452Ter/p.Val1557fs
#AML_EC_68	64	1	Dead	0	p.Arg882His	p.Trp288fs			
#AML_EC_69	41	1	Alive	1	p.Trp795Arg				
#AML_EC_7	56	1	Dead	1	p.Arg882Cys				p.Asn715Asp/p.Cys1273Ser
#AML_EC_70	53	1	Dead	0					
#AML_EC_71	47	1	Alive	NA	p.Arg882Cys		p.Ile2780Thr		
#AML_EC_72	50	2	Alive	0	p.Arg320Ter	p.Trp288fs			
#AML_EC_73	55	2	Dead	0					
#AML_EC_74	54	1	Dead	1					
#AML_EC_75	75	1	Dead	1	p.Tyr533Cys				
#AML_EC_76	32	2	Alive	0					
#AML_EC_78	52	1	Alive	0		p.Trp288fs		p.Pro599Leu	
#AML_EC_79	60	1	Dead	0					
#AML_EC_80	75	1	Dead	0	p.Asn810fs				
#AML_EC_81	37	2	Alive	1	p.Pro385fs	p.Trp288fs			
#AML_EC_82	78	2	Dead	0					
#AML_EC_84	58	2	Alive	0					
#AML_EC_85	47	2	Dead	1	p.Ser663Leu/	p.Trp288fs	p.Thr3756Pro	p.Arg685Cys	
#AML_EC_86	76	2	Alive	0		p.Trp288fs			
#AML_EC_87	28	2	Alive	0		p.Trp288fs		p.Pro2618Leu/p.Ser237Pro	
#AML_EC_88	20	1	Alive	0					
#AML_EC_89	74	2	Dead	0	p.Leu547Phe		p.Gln3225Lys|		p.Leu1816Ter
#AML_EC_9	57	1	Alive	0		p.Trp288fs			
#AML_EC_90	71	1	Dead	NA	p.Arg882His	p.Trp288fs			
#AML_EC_91	65	2	Dead	0					
#AML_EC_92	51	2	Alive	0		p.Trp288fs			
#AML_EC_93	48	1	Alive	0					
#AML_EC_94	50	1	Alive	1		p.Trp288fs			
#AML_EC_96	59	1	Dead	1	p.Arg882Cys				
#AML_EC_97	26	2	Alive	0					
#AML_EC_98	45	2	Alive	0	p.Arg882His			p.Ala4Pro	
#AML_EC_99	39	1	Alive	0			p.Ile1360Thr/p.Thr3756Pro		

Patient ID	FCGBP	ASXL1	FLT3	RUNX1	CEBPA	PKD1L2	PLA1A	CSMD1
#AML_EC_12								
#AML_EC_25								
#AML_EC_29								
#AML_EC_64								
#AML_EC_83		p.Gly646fs	p.Asp835Tyr/p.Asn676His				p.Ala270Thr	
#AML_EC_4	p.Thr1432Pro		p.Asp835Tyr					
#AML_EC_56	p.Tyr52Ser				p.Asn356fs			
#AML_EC_8	p.Thr1432Pro		p.Asp835Tyr					
#AML_EC_95		p.Met1492Val/p.Asp720fs				p.Val878Leu		
#AML_EC_49			p.Asp835Glu					p.His75Arg
#AML_EC_22	p.Leu4907Phe	p.Ala619fs		p.His105Pro				
#AML_EC_36	p.Val1123Met							
#AML_EC_62	p.Pro4620Ser				p.Gly36fs			
#AML_EC_31	p.Val4201Gly	p.Glu676fs						
#AML_EC_11							p.Asn395Lys	
#AML_EC_77								
#AML_EC_1		p.Gly646fs		p.Arg320Ter				
#AML_EC_10	p.Gln1583Ter							
#AML_EC_10(						p.Ile539Val		
#AML_EC_13								
#AML_EC_14			p.Tyr572Cys/p.Ala848Pro/p.Ala680Val					
#AML_EC_15							p.Asn405Lys	
#AML_EC_16				p.Arg145Gly			p.Thr107Asn	
#AML_EC_17	p.Thr15Asn			p.Ser322Ter				
#AML_EC_18	p.Pro4686Ser/p.Val357Met		p.Val194Met					
#AML_EC_19		p.Gly646fs		p.Arg320Ter		p.Gly813Val	p.Asn395Lys	
#AML_EC_2								p.Val3494Gly
#AML_EC_20					p.Leu317_Thr318insMet			
#AML_EC_21					p.Leu317_Thr318insMet			p.Ile2443Val
#AML_EC_23			p.Lys663Gln					
#AML_EC_24						p.Tyr760Cys		
#AML_EC_26					p.Gly248fs			p.Leu715Val
#AML_EC_27	p.Met89Val	p.Lys580Ter						
#AML_EC_28				p.Arg162Gly			p.Val229Ile	
#AML_EC_3								p.Val3494Gly
#AML_EC_30					p.Gln41fs	p.Arg961Gln	p.Ser444Thr	
#AML_EC_32								p.Tyr845Ser
#AML_EC_33								
#AML_EC_34			p.Tyr591Asp					
#AML_EC_35								
#AML_EC_37			p.Asp835Tyr					
#AML_EC_38								
#AML_EC_39			p.Asp835His				p.Asn395Lys	
#AML_EC_40	p.Ala1343Val/p.Ile4	p.Ile552Val/p.Ser1231Phe		p.Asn139Lys/p.Cys99fs				
#AML_EC_41					p.Glu59fs			
#AML_EC_42		p.Gly646fs					p.Asn395Lys	
#AML_EC_43								
#AML_EC_44								
#AML_EC_45								
#AML_EC_46							p.Asn395Lys	
#AML_EC_47								
#AML_EC_48								
#AML_EC_5	p.Val4201Gly	p.Gly646fs						
#AML_EC_50		p.Ala1312Val						p.Ile2443Val
#AML_EC_51		p.Glu1102Asp			p.Trp288fs			
#AML_EC_52					p.His24fs	p.Asn236Ser		
#AML_EC_53								
#AML_EC_54	p.Tyr872Cys					p.Arg961Gln		
#AML_EC_55			p.Asp835Tyr					
#AML_EC_57		p.Glu1102Asp			p.Gly32Val|			
#AML_EC_58								
#AML_EC_59								
#AML_EC_6	p.Tyr872Cys							
#AML_EC_60							p.Val426Ile	
#AML_EC_61		p.Gly646fs				p.Asn236Ser		
#AML_EC_63				p.Arg204Ter		p.Arg961Gln		
#AML_EC_65	p.Arg1266His		p.Tyr589Ter					
#AML_EC_66					p.Gln41fs			
#AML_EC_67								
#AML_EC_68	p.Thr15Asn		p.Gln580Glu					
#AML_EC_69								
#AML_EC_7								
#AML_EC_70		p.Gln512Ter/p.Glu477Gln				p.Asn236Ser		
#AML_EC_71			p.Val592Ala/p.Asn841Ile					
#AML_EC_72			p.Asp835His					
#AML_EC_73		p.Gly1397Ser/p.Ter1542fs		p.Phe163Leu		p.Arg961Gln		
#AML_EC_74								
#AML_EC_75								
#AML_EC_76								
#AML_EC_78	p.Ala1343Val		p.Asn676Lys					
#AML_EC_79								
#AML_EC_80								
#AML_EC_81	p.Ala1343Val							
#AML_EC_82								
#AML_EC_84				p.Leu112fs				p.Ala1328Val
#AML_EC_85								
#AML_EC_86								p.Leu3443Pro/p.Tyr2768Cys
#AML_EC_87								
#AML_EC_88		p.Thr936Pro			p.Glu309_Thr310insLys			
#AML_EC_89			p.Val592Ala	p.Ser37Ile	p.Trp288fs			
#AML_EC_9		p.Gly646fs						
#AML_EC_90	p.Lys1328Gln	p.Met1249Val	p.Phe594Tyr					p.Ser1066Phe
#AML_EC_91				p.Arg107Ser				
#AML_EC_92								
#AML_EC_93								p.Pro2261Ala
#AML_EC_94								
#AML_EC_96							p.Asn395Lys	
#AML_EC_97								
#AML_EC_98	p.Lys5355Glu							
#AML_EC_99				p.Ala60Val				

Patient ID	EPX	ZNF234	GATA2	CIC	BCOR	WT1	FAM69B	NLRP5	IDH2
#AML_EC_12	p.Arg713Gln								
#AML_EC_25									
#AML_EC_29			p.Pro161Ala						
#AML_EC_64									
#AML_EC_83	p.Arg286His/p.Cys257Ter			p.Pro909Leu					
#AML_EC_4									
#AML_EC_56			p.His127Pro						
#AML_EC_8									
#AML_EC_95									
#AML_EC_49		p.Leu130Phe							
#AML_EC_22									
#AML_EC_36									
#AML_EC_62	p.Leu335Met	p.Ile119Leu							
#AML_EC_31					p.Arg1532Gly				
#AML_EC_11			p.Leu321Phe	p.Arg1515Gly					
#AML_EC_77									
#AML_EC_1							p.Thr93Pro		
#AML_EC_10									
#AML_EC_10(									
#AML_EC_13									
#AML_EC_14			p.His460Pro	p.Thr421Pro					
#AML_EC_15			p.His460Pro						
#AML_EC_16									
#AML_EC_17	p.Gly680Ser		p.Asn317Ser/p.Gly100Val		p.Arg810Ter				
#AML_EC_18	p.Gly680Ser							p.Ala472Thr	
#AML_EC_19									
#AML_EC_2							p.Thr122Pro		p.Thr352Pro
#AML_EC_20		p.Leu130Phe		p.Ala774Val		p.Tyr339Ter			
#AML_EC_21									
#AML_EC_23		p.Gly602Glu				p.Arg434Pro			
#AML_EC_24						p.Val379fs			
#AML_EC_26								p.Arg542Lys	
#AML_EC_27	p.Arg364His				p.Leu686Ter				
#AML_EC_28		p.Cys324Arg							
#AML_EC_3							p.Thr122Pro		
#AML_EC_30	p.Leu335Met	p.Ala361Asp							
#AML_EC_32									
#AML_EC_33									
#AML_EC_34									
#AML_EC_35								p.Arg518Cys	
#AML_EC_37			p.His460Pro		p.Asn575Ser				
#AML_EC_38	p.Phe308Leu								p.Arg140Gln
#AML_EC_39	p.Gly680Ser								
#AML_EC_40		p.Gly602Glu	p.His460Pro			p.Gln366Ter			
#AML_EC_41						p.Gly257Asp			
#AML_EC_42							p.Thr93Pro		p.Arg172Lys
#AML_EC_43									
#AML_EC_44									
#AML_EC_45									
#AML_EC_46									
#AML_EC_47									p.Arg140Gln
#AML_EC_48									p.Arg140Gln
#AML_EC_5	p.Leu335Met	p.Cys598Phe							p.Arg140Gln
#AML_EC_50				p.Ter1609Gly	p.Asn1511fs				
#AML_EC_51									
#AML_EC_52	p.Cys257Ter		p.His460Pro				p.Thr93Pro		
#AML_EC_53									
#AML_EC_54									
#AML_EC_55		p.Ser217Leu							
#AML_EC_57									p.Arg140Gln
#AML_EC_58						p.Val379fs			
#AML_EC_59								p.Arg542Lys	
#AML_EC_6					p.Gln231His	p.Arg458Pro			p.Arg140Gln
#AML_EC_60									
#AML_EC_61									
#AML_EC_63									
#AML_EC_65						p.Val371Glu			
#AML_EC_66			p.Trp360Leu						
#AML_EC_67									
#AML_EC_68									
#AML_EC_69									p.Arg140Gln
#AML_EC_7						p.Arg434Cys			
#AML_EC_70	p.Phe308Leu						p.Leu247fs	p.Ala472Thr	
#AML_EC_71									
#AML_EC_72									
#AML_EC_73									
#AML_EC_74							p.Cys14Tyr		
#AML_EC_75									
#AML_EC_76									
#AML_EC_78			p.His460Pro	p.Arg30His				p.Ala472Thr	
#AML_EC_79	p.Cys257Ter		p.Arg362Gln						
#AML_EC_80								p.Ala472Thr	
#AML_EC_81		p.Leu130Phe							
#AML_EC_82									
#AML_EC_84		p.Cys598Phe			p.Arg1547Ter			p.His863Pro	
#AML_EC_85									
#AML_EC_86			p.His460Pro						
#AML_EC_87									
#AML_EC_88						p.Ala211fs		p.Ser586Thr	
#AML_EC_89									
#AML_EC_9									
#AML_EC_90									
#AML_EC_91					p.Asp1627As	p.Arg380Gly/p.Arg380fs			
#AML_EC_92									
#AML_EC_93								p.Arg562His	
#AML_EC_94									
#AML_EC_96			p.Lys378Glu						
#AML_EC_97									
#AML_EC_98			p.His460Pro	p.Ter1609Gly					p.Arg172Lys
#AML_EC_99						p.Val379fs			

Patient ID	NRAS	KRAS	PTPN11	NSD1	STAG2	ERG	ZNF253	ACTN2	TP53	PHF6	RAD21	IDH1
#AML_EC_12				p.Val1882Gly		p.Ser13Asn						
#AML_EC_25			p.Ala72Thr	p.Asn2445Tyr								
#AML_EC_29				p.Leu2617Ser								
#AML_EC_64	p.Gln61His			p.Ser1241Thr								
#AML_EC_83				p.Tyr1971Cys								
#AML_EC_4				p.Val1882Gly	p.Gln140Ter							
#AML_EC_56				p.Val1882Gly								
#AML_EC_8				p.Val1882Gly	p.Gln140Ter							
#AML_EC_95				p.Val2296Ala								
#AML_EC_49			p.Asp61Tyr									
#AML_EC_22					p.Phe468fs							
#AML_EC_36					p.Asp850Glu							
#AML_EC_62												
#AML_EC_31	p.Gly12Asp											p.Gly402Ter
#AML_EC_11												
#AML_EC_77												
#AML_EC_1	p.Gly13Asp|				p.Met1126fs							
#AML_EC_10					p.Gly257Glu							
#AML_EC_10(												p.Arg132Gly
#AML_EC_13												
#AML_EC_14												
#AML_EC_15												
#AML_EC_16												
#AML_EC_17												
#AML_EC_18												
#AML_EC_19												
#AML_EC_2											p.Val253Gly	
#AML_EC_20						p.Gly262Ser						
#AML_EC_21			p.Ala72Ser									p.Arg132His
#AML_EC_23												
#AML_EC_24												
#AML_EC_26												p.Arg132Gly
#AML_EC_27	p.Gln61Arg											
#AML_EC_28	p.Gly12Asp											
#AML_EC_3											p.Val253Gly	
#AML_EC_30											p.Glu614Ter	
#AML_EC_32		p.Gly12Asp	p.Asp61Tyr									
#AML_EC_33	p.Gly12Asp											
#AML_EC_34												
#AML_EC_35												
#AML_EC_37		p.Gln61Pro										
#AML_EC_38												
#AML_EC_39			p.Asp61Tyr									
#AML_EC_40												
#AML_EC_41												
#AML_EC_42												
#AML_EC_43												
#AML_EC_44												
#AML_EC_45			p.Ser502Leu									
#AML_EC_46	p.Gly12Asp											
#AML_EC_47												
#AML_EC_48												
#AML_EC_5		p.Gly12Val	p.Gly503Ala						p.Cys277Tyr			
#AML_EC_50												
#AML_EC_51		p.Ala146Thr										
#AML_EC_52												
#AML_EC_53												
#AML_EC_54		p.Gly13Asp/p.Lys117Arg										
#AML_EC_55												
#AML_EC_57												
#AML_EC_58			p.Glu76Lys									
#AML_EC_59			p.Ala72Val									
#AML_EC_6												
#AML_EC_60												p.Arg132Gly
#AML_EC_61												
#AML_EC_63												
#AML_EC_65												
#AML_EC_66												
#AML_EC_67												
#AML_EC_68	p.Gln61Arg											
#AML_EC_69												
#AML_EC_7												
#AML_EC_70												p.Arg132Cys
#AML_EC_71												
#AML_EC_72												
#AML_EC_73					p.Cys347Ter		p.Ile329Thr					
#AML_EC_74												
#AML_EC_75												
#AML_EC_76												
#AML_EC_78												
#AML_EC_79												
#AML_EC_80		p.Gly12Ser										
#AML_EC_81												
#AML_EC_82												
#AML_EC_84		p.Gly12Asp										
#AML_EC_85												
#AML_EC_86												p.Arg132Ser
#AML_EC_87												
#AML_EC_88						p.His434Pro						
#AML_EC_89												
#AML_EC_9												
#AML_EC_90												
#AML_EC_91		p.Gly12Val										
#AML_EC_92												
#AML_EC_93												
#AML_EC_94												
#AML_EC_96												
#AML_EC_97												
#AML_EC_98												
#AML_EC_99												

PTGIS	KIT	FAM5C	HK2	PARD3B	TSPAN5	IGF1	EZH2	KDM6A	SUV39H1	CHD4	SMC1A	SMC3	PCMTD1	GIT1	GNAS	OR8H3	SIPA1L2	GPR6	PLCE1	COL12A1	KCNU1	STC2	ATP6AP1	BTNL3	CHI3L2	DGCR2	DLG1	PCDHA7	TRIM67	VPS37C	ZCCHC14	ZMYND17	ZSWIM1	GRID1	HYDIN	MAGI1	TEKT4
			p.Gly666Ser																				p.Gly107Arg						p.His163Pro								
																		p.Ala18Ser																p.Ala588Asp			
																													p.Arg584Gln								
							p.Asn546Lys																												p.Asp3880Asn/p.Glu2551Gly		
																												p.Pro576Arg									
																			p.Ala1045Ser																		
			p.Pro736Leu																p.Asp1442Gly																		
	p.Val530Ile																		p.Asp308Glu		p.Ile427Thr													p.Arg462Leu			
																	p.Glu1295Gly		p.Leu219Val	p.Ile1068Val																p.Tyr624Cys	
																			p.Pro1890Leu		p.Arg704Ter																
			p.Val633Met																p.Thr1078Pro/p.Asn1573Thr																p.Ile4944Leu		
																	p.Val1508Ala		p.Val378Leu															p.Ala588Asp			
				p.Asp736Glu			p.Asp677Gly																														
							p.Ile631Val													p.Pro2277Gln/p.Ser791Tyr														p.Ala588Asp			
								p.Arg658Ter																													
	p.Pro114Ala									p.Arg813Cys																p.Arg491Pro											
								p.Cys1234Gly						p.Thr66Met										p.Thr402Pro													
							p.Ser624Ala																														
																				p.Val2428Gly																p.Asp254Val	
																						p.Met86Ile															
												p.Leu1127Arg																						p.Ala588Asp			
				p.Ala155Thr/p.Ser366Leu				p.Ala1036Val																													
																		p.Ala18Ser				p.Ser101Leu															
	p.Glu514fs						p.Val626Met											p.Ala18Ser									p.Asp267Gly										
										p.Lys67Glu																											
																				p.Val2428Gly																p.Arg1388_Arg1395del	
															p.Ser285Phe																						
							p.Tyr701Cys					p.Asn616Ser																							p.His3551Arg		
				p.Asn561Asp																																	
																p.Pro137Ser	p.Val113Ile																				
												p.Arg381Gln																									
																p.Pro137Ser												p.Thr680Met							p.His3551Arg		
																													p.Gly273Asp	p.Gly295Asp							
				p.Asp1030Asn								p.Arg391Gly																									
																																		p.Ala588Asp			
p.Arg91Cys																																					
p.Arg91Cys																																					
			p.Arg497Gln																																	p.His261Arg	
																											p.Val739Leu		p.Gly293Ala								
								p.Cys1234Gly									p.Phe21Leu																				
			p.Pro736Leu																																		
						p.Gly194Glu										p.Thr95Met															p.Thr199Ile						
				p.Thr125Asn/p.Val313Ile											p.Glu718Lys																						
																											p.Arg716Cys										
																																				p.Gln412_Gln415del	
										p.Lys577Thr	p.Lys768del																							p.Ala588Asp		p.Ala1221Thr	
											p.Gln771Arg										p.Ser660Gly																
																																		p.Ala588Asp	p.His3551Arg		
																	p.Arg798Gln																				
							p.Asp730Ter			p.Tyr892Ter														p.Gly339Val					p.Gly293Ala								
																p.Pro137Ser				p.Gly899Glu																	
												p.Arg827Gln																									
				p.Asp995Val														p.Tyr329Cys							p.Thr317Met				p.Pro595Ser								
											p.Arg699Cy/p.Arg711Gln																										
																	p.Asp1088Asn				p.Pro655Leu																
								p.Cys468Arg				p.Gly667Cys																									
			p.His56Pro																																		
																													p.Gly273Asp								
																	p.Arg1167Gly																				
p.Arg275Gln	p.Val852Ile							p.Cys1234Gly																													
								p.Cys1234Gly																													
																																				p.Leu1337Val	
																p.Pro137Ser																					
																																			p.Leu4167Phe		

FAM70B	*U2AF1*	*HNRNPK*	*CCNL2*	*INTS12*	*NFATC2*	*ZCCHC16*	*CD22*	*NEDD9*	*PIK3R1*	*EPHB1*	*USP9X*	*USP34*	*C12orf51*	*OTUD7A*
								p.Ser387Tyr	p.Arg358Leu			p.Glu1246Lys		
								p.Gln782His						
	p.Gln157Arg													
			p.Thr320Ile											
												p.Arg3096Gln		
										p.Thr981Met				
					p.Val51Leu									
										p.Thr981Met	p.Ile1974Val			
	p.Glu159_Met160insTyrGlu										p.Ile535fs			
												p.Ala1569Val		
	p.Gln157Arg													
			p.Trp262Ter											
					p.Gln902Pro									
														p.Lys247Arg
											p.Arg546Ile			
			p.Arg497His											
							p.Thr793Met							
												p.His1638Arg		
	p.Ser34Phe				p.Met832Ile					p.Thr981Met				
					p.Trp399Cys									
									p.Arg409Gln	p.Asn430Thr				
