# Supplementary figures and images for: The modular network structure of the mutational landscape of Acute Myeloid Leukemia
Source: PLoS One. 2018 Oct 10;13(10):e0202926. doi: 10.1371/journal.pone.0202926 (PMC6179200; doi:10.1371/journal.pone.0202926)

S1 Fig.

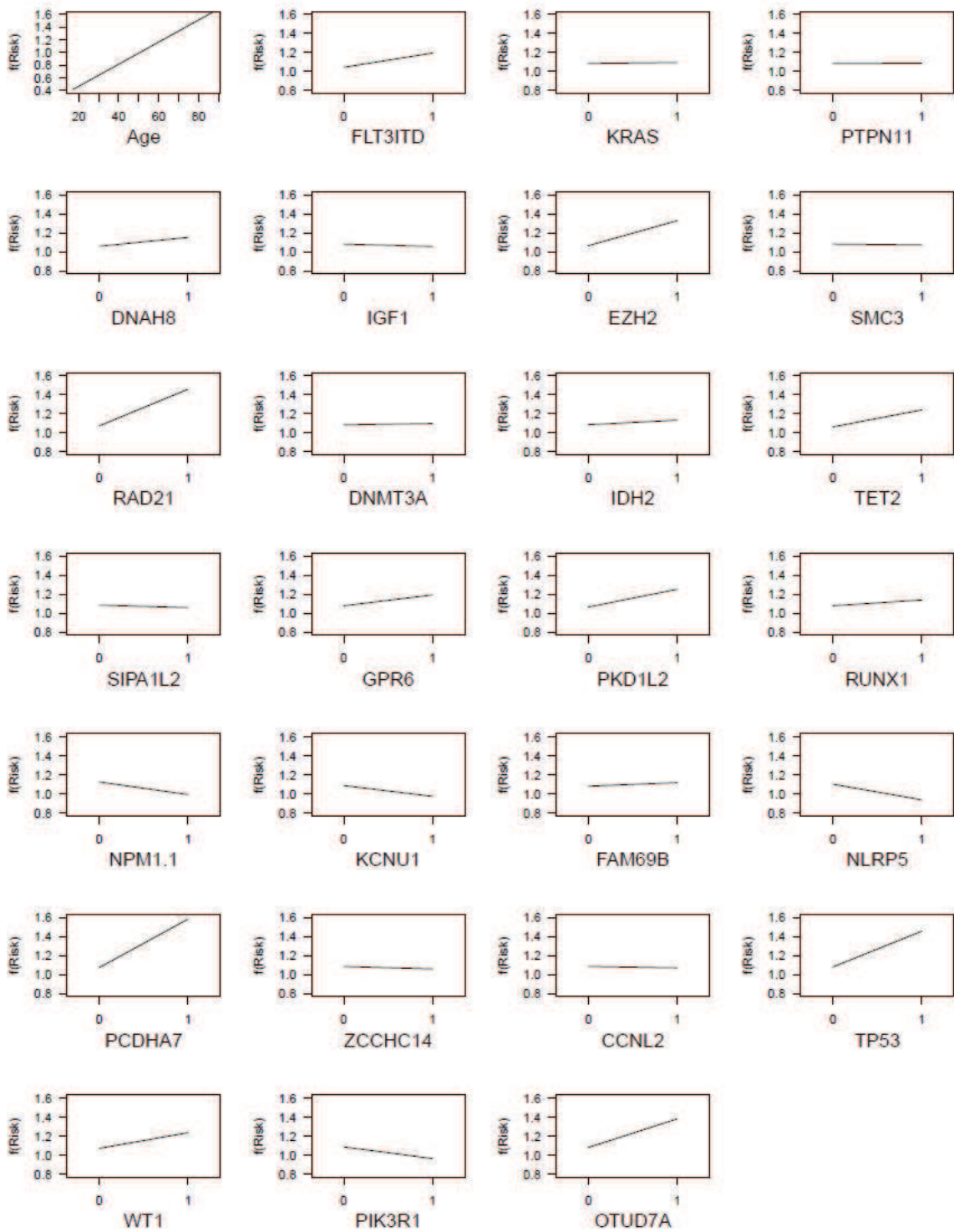

Supplement: S1 Fig — (PDF) [file pone.0202926.s007.pdf]

S2 Fig

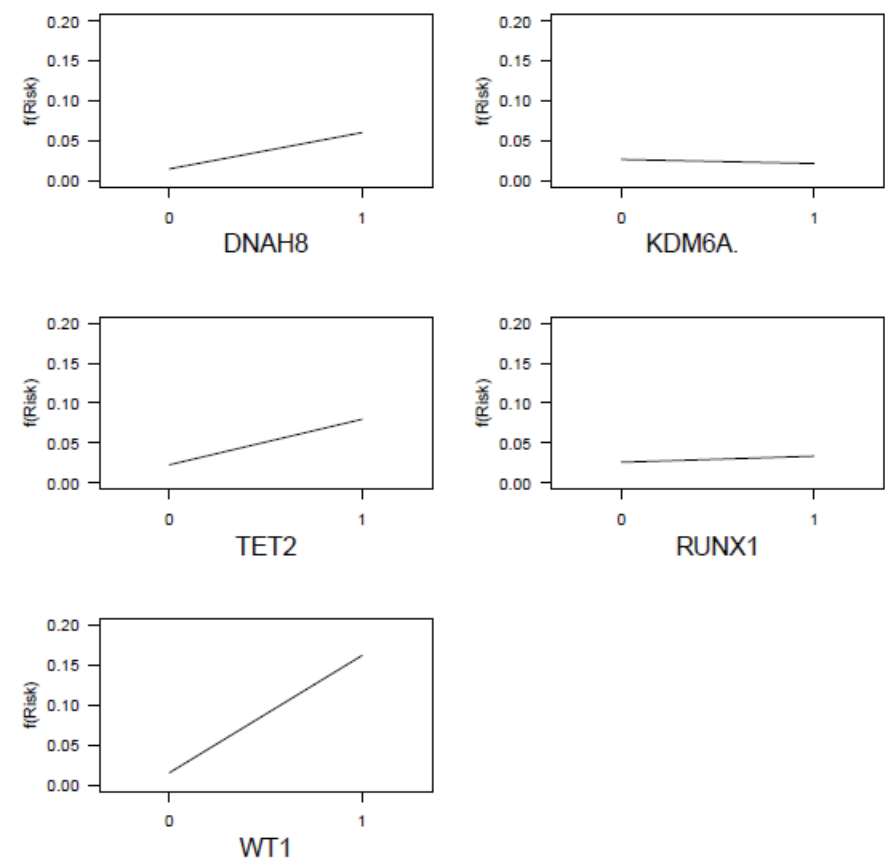

Supplement: S2 Fig — (PDF) [file pone.0202926.s008.pdf]
